# Independent Increments and Group Sequential Tests

**DOI:** 10.1002/sim.70307

**Published:** 2025-11-07

**Authors:** Anastasios A. Tsiatis, Marie Davidian

**Affiliations:** ^1^ Department of Statistics North Carolina State University Raleigh North Carolina USA

**Keywords:** censored survival analysis, group sequential test, independent increments, restricted mean survival time, Wilcoxon test

## Abstract

Widely used methods and software for group sequential tests of a null hypothesis of no treatment difference that allow for early stopping of a clinical trial depend primarily on the fact that sequentially‐computed test statistics have the independent increments property. However, there are many practical situations where the sequentially‐computed test statistics do not possess this property. Key examples are in trials where the primary outcome is a time to an event but where the assumption of proportional hazards is likely violated, motivating consideration of treatment effects such as the difference in restricted mean survival time or the use of approaches that are alternatives to the familiar logrank test, in which case the associated test statistics may not possess independent increments. We show that, regardless of the covariance structure of sequentially‐computed test statistics, one can always derive linear combinations of these test statistics sequentially that do have the independent increments property. We also describe how to best choose these linear combinations to target specific alternative hypotheses, such as proportional or non‐proportional hazards or log odds alternatives. We thus derive new, sequentially‐computed test statistics that not only have the independent increments property, supporting straightforward use of existing methods and software, but that also have greater power against target alternative hypotheses than do procedures based on the original test statistics, regardless of whether or not the original statistics have the independent increments property. We illustrate with two examples.

## 1 Introduction

Interim monitoring of clinical trials to allow for the possibility of early stopping for efficacy or futility is a critically important practice in health sciences and biopharmaceutical research and is ordinarily accomplished through the use of group sequential methods. Key to straightforward implementation of such methods is the property of independent increments of sequentially‐computed test statistics; see Kim and Tsiatis [1] for a comprehensive overview. Specifically, computation of sequential stopping boundaries to which the test statistic is compared at interim analyses requires multivariate integration with respect to the (usually asymptotic) joint distribution of the test statistic across monitoring times. However, if the increments between statistics used to construct the test statistics are independent, the computation simplifies to involve only simple univariate integration, which is the foundation for the widely used group sequential methods [2, 3, 4] that are available in standard software.

In many trials, inference focuses on a treatment effect that can be characterized by a single parameter in a relevant statistical model, for example, the difference of treatment means in the case of a continuous outcome or the risk difference, risk ratio, or odds ratio with a binary outcome, where the definition of the parameter is the same across all time. Under these conditions, it has been shown [5, 6] that efficient estimators of such treatment effect parameters, computed sequentially, have the independent increments property, so that standard group sequential methods are readily implemented. However, there are many situations where efficient test procedures may be unavailable or not straightforward to implement. For example, Van Lancker et al. [7] discuss use of a test statistic based on an estimator for the treatment effect parameter that incorporates covariate adjustment for prespecified baseline covariates thought to be prognostic for the outcome. Although covariate adjustment can enhance the precision of the final analysis, owing to the complexity of the efficient estimator, the estimator and thus statistic the authors adopt for practical use is not efficient and thus does not enjoy the independent increment structure, so that standard group sequential methods cannot be used.

The independent increments property also may not hold because of the nature of the treatment effect. In clinical trials in chronic disease and especially in cancer, the primary outcome is a time to an event, and the most common characterization of treatment effect is through the hazard ratio under the assumption of proportional hazards, with the primary analysis typically based on the logrank test or Cox model. However, the proportional hazards assumption is often violated, which has led to interest in representing the treatment effect through the difference in restricted mean survival time (RMST), the mean time to the event restricted to a specified time L [8]. As discussed by Murray and Tsiatis [9] and Lu and Tian [10], because of limited follow up, at the jth interim analysis at time $t_{j}$, say, the parameter of interest is the difference in RMST to a time $L_{j}\leq t_{j}$; thus, the parameter of interest changes over time, and it can be shown that the independent increments property does not hold for the corresponding statistic.

Test statistics that are motivated by intuitive considerations may or may not possess the independent increments property. For example, an alternative approach when violation of the proportional hazards assumption is thought to be due to differences that manifest early in time is to base the analysis on Gehan's Wilcoxon test [11], which gives more weight to events at early time points. Here, the null hypothesis of a difference in event time distributions cannot be expressed in terms of a single parameter, and the statistic on which this test is based has been shown to not possess the independent increments property [12]. In contrast, that for the logrank test, which can also be motivated from an intuitive perspective, does have independent increments.

In this article, we show that, regardless of the covariance structure of sequentially‐computed statistics, and thus regardless of whether or not they possess the independent increments property, it is always possible to derive sequential linear combinations of these statistics that do possess this property. Thus, for a given statistic, a group sequential test procedure based on these linear combinations can be implemented using standard methods and software. If the original statistics are not efficient, our formulation shows that linear combinations of them leading to independent increments can be chosen judiciously to also result in more efficient tests. As a result, depending on the alternative of interest, basing interim monitoring on tests involving such linear combinations of statistics may yield stronger evidence for early stopping, even if the original statistics possess the independent increments property. In Section 2, we present the general statistical framework and the main result. In Section 3, we show how the result is used to choose linear combinations to yield independent increments and increase efficiency. We consider two examples in Section 4: interim monitoring based on Gehan's Wilcoxon test when the treatment effect is the difference in treatment‐specific survival distributions, and interim monitoring when the treatment effect is the difference in treatment‐specific RMST. We discuss practical implementation in Section 5 and demonstrate the methods by retroactive application to a completed cancer clinical trial in Section 6.

## 2 Statistical Framework and Main Result

The property of independent increments can be characterized as follows. Consider a normally distributed random vector $Z={\left(Z_{1},\ldots ,Z_{K}\right)}^{T}$ with (K×K) covariance matrix ${𝒱}_{Z}$. Then Z has the independent increments property if ${𝒱}_{Z}(j,k)={𝒱}_{Z}(j,j)$, j≤k, j,k=1,…,K, where ${𝒱}_{Z}(j,k)$ denotes the (j,k)th element of ${𝒱}_{Z}$.

We now state and prove the general result that serves as the foundation for the methodology. To this end, let $X_{1},\ldots ,X_{K}$ denote time ordered, sequentially‐computed random variables. Later, these variables will be (suitably normalized) statistics computed at K possible interim analyses of a clinical trial using all the available data through each interim analysis. Often, such statistics are asymptotically normally distributed and constructed so that they have mean zero under the relevant null hypothesis of no treatment effect. Let $X={\left(X_{1},\ldots ,X_{K}\right)}^{T}$ be the K random variables collected into a vector, and, for j=1,…,K, define the (K×1) vector ${\underline{X}}_{j}={\left(X_{1},\ldots ,X_{j},0,\ldots ,0\right)}^{T}$; that is, the K‐dimensional vector leading with ${\left(X_{1},\ldots ,X_{j}\right)}^{T}$ followed by (K−j) zeros. Denote the (K×K) covariance matrix of X by 𝒱 and the covariance matrix of ${\underline{X}}_{j}$ by ${𝒱}_{j}$, the (K×K) matrix with upper left (j×j) submatrix equal to the covariance matrix of ${\left(X_{1},\ldots ,X_{j}\right)}^{T}$ and all remaining elements equal to zero.

For j=1,…,K, let $a_{j}={\left(a_{j1},\ldots ,a_{jj},0,\ldots ,0\right)}^{T}$ be a K‐dimensional vector, where the first j components $a_{j1},\ldots ,a_{jj}$ are constants followed by (K−j) zeros. Then denote the sequentially‐computed linear combinations of X by

(1) $$Y={\left(Y_{1},\ldots ,Y_{K}\right)}^{T},\;Y_{1}=a_{1}^{T}X,\ldots ,Y_{K}=a_{K}^{T}X.$$

Henceforth, assume that the covariance matrix 𝒱 has full rank and thus a unique inverse ${𝒱}^{-1}$ and that the linear combinations in (1) are nontrivial in the sense that $Y_{j}=a_{j1}X_{1}+\ldots +a_{jj}X_{j}$, which in the context of interim monitoring would be the linear combination computed at the jth interim analysis, must include $X_{j}$; that is, $a_{jj}\neq 0$, j=1,…,K. This condition guarantees that the covariance matrix of Y also has full rank. The key result, that these sequentially‐computed linear combinations of X have the independent increments property, follows from Theorem 1, the proof of which is presented in the Supporting Information.

Theorem 1 *Under the above conditions, with* 𝒱 *of full rank and* ${𝒱}_{j}$, j=1,…,K, *defined as above, the vector of nontrivial, sequentially‐computed linear combinations* $Y={\left(Y_{1},\ldots ,Y_{K}\right)}^{T}$ *has the independent increments property if and only if there exists a vector of constants* $b={\left(b_{1},\ldots ,b_{K}\right)}^{T}$ *such that* $a_{j}={𝒱}_{j}^{-}{\underline{b}}_{j}$, *where* ${\underline{b}}_{j}={\left(b_{1},\ldots ,b_{j},0,\ldots ,0\right)}^{T}$, j=1,…,K, *and* ${𝒱}_{j}^{-}$ *is the* (K×K) *matrix with upper left hand* (j×j) *submatrix the inverse of the covariance matrix of* ${\left(X_{1},\ldots ,X_{j}\right)}^{T}$ *and all remaining elements of the matrix equal to zero; that is*, ${𝒱}_{j}^{-}$ *is the Moore‐Penrose generalized inverse of* ${𝒱}_{j}$.

## 3 Derivation of Test Procedures

### 3.1 Choice of Linear Combination

Theorem 1 is a very general result, demonstrating that linear combinations of time ordered random variables $X={\left(X_{1},\ldots ,X_{K}\right)}^{T}$ with covariance matrix 𝒱 will have the independent increments property if the coefficients of the linear combination satisfy

$$a_{j}={𝒱}_{j}^{-}{\underline{b}}_{j},\text{where}{\underline{b}}_{j}={\left(b_{1},\ldots ,b_{j},0\ldots ,0\right)}^{T}$$

for some arbitrary vector $b={\left(b_{1},\ldots ,b_{K}\right)}^{T}$. Consequently, to construct a linear combination of elements of X with independent increments, one can choose any arbitrary K‐dimensional vector of constants b. However, depending on the objective, judicious choices of b can be identified.

We now discuss considerations for the choice of b when interest focuses on a null hypothesis $H_{0}$ of no treatment effect to be tested in a clinical trial potentially involving n subjects at up to K interim analyses. Let $X_{n}={\left(X_{1,n},\ldots ,X_{K,n}\right)}^{T}$ be the vector of suitably normalized statistics used to form the test statistic for this hypothesis, to be computed sequentially over time. For example, if the test statistic is the score test statistic based on a suitable log likelihood, $X_{j,n}$ is equal to $n^{-1/2}$ times the score. We emphasize that the test statistic computed in practice at the jth interim analysis at time $t_{j}$ is based only on the data accumulated through $t_{j}$ and does not involve n; we discuss this further below. We consider normalized statistics $X_{j,n}$ solely for the theoretical developments that support how b should be chosen. Here, taking n to grow large (n→∞) can be viewed as allowing the staggered entry times at which subjects enroll in the trial to become more and more dense. The statistics can be based on parametric, semiparametric, or nonparametric models and, under $H_{0}$, generally satisfy $X_{n}\overset{D}{\to }N(0,𝒱)$ as n→∞; that is, $X_{n}$ converges in distribution to a normal random vector with mean zero and covariance matrix 𝒱, so that, for n large,

$$X_{n}\overset{\cdot }{∼}N(0,𝒱)\text{under}H_{0}.$$

Letting ${\hat{𝒱}}_{n}$ be a consistent estimator for 𝒱, typically, at the jth interim analysis, one computes the standardized test statistic

$$X_{j,n}/{\left\{{\hat{𝒱}}_{n}(j,j)\right\}}^{1/2},$$

which thus has an approximate N(0,1) distribution under $H_{0}$, and $H_{0}$ is rejected if the test statistic (or absolute value thereof) exceeds some critical value. The standard approach to identifying the critical value is through a specified α‐spending function [4] that, for chosen level of significance α, determines how α is to be “spent” over the potential K analyses. More precisely, the α‐spending function is a sequence $\alpha_{1}<\cdots <\alpha_{K}=\alpha$ such that the probability of rejecting $H_{0}$ at or before the jth interim analysis is $\alpha_{j}$. The corresponding critical values are referred to as the stopping boundaries. In general, $X_{n}$ may or may not have the independent increments property (asymptotically).

Ordinarily, the goal is for a test procedure to have adequate power to detect some alternative hypothesis of interest. Typically, under a fixed alternative $H_{A}$, common test procedures have the property that power converges to one as n→∞, so that usual asymptotic theory yields no insight into practical performance. Thus, to achieve nontrivial results reflecting the large sample properties of a test, rather than consider a fixed alternative, a standard approach is to consider performance of the test under a sequence of so‐called local alternatives $H_{A,n}$, which are such that the alternative hypothesis $H_{A,n}$ approaches the null $H_{0}$ as n→∞. As ordinarily the case in such local power analysis, with fixed alternatives characterized by a quantity δ that equals zero under $H_{0}$, as in the examples we consider later in Section 4, the alternative under $H_{A,n}$ is $\delta_{n}$, say, where $n^{1/2}\delta_{n}\to \tau$ as n→∞. Under such local alternatives, it can be shown that $X_{n}\overset{D}{\to }N(\mu ,𝒱)$ for some K‐dimensional vector $\mu ={\left(\mu_{1},\ldots ,\mu_{K}\right)}^{T}$ depending on τ, where 𝒱 is the same covariance matrix as under $H_{0}$, so that

$$X_{n}\overset{\cdot }{∼}N(\mu ,𝒱)\text{under}H_{A,n}.$$

Thus, to derive the optimal linear combination of $X_{1,n},\ldots ,X_{j,n}$ yielding the greatest power to detect the alternative $H_{A,n}$ at the jth interim analysis, consider the standardized test statistic

$${\underline{c}}_{j}^{T}{\underline{X}}_{j,n}/{\left({\underline{c}}_{j}^{T}{𝒱}_{j}{\underline{c}}_{j}\right)}^{1/2},$$

where ${\underline{c}}_{j}={\left(c_{j1},\ldots ,c_{jj},0,\ldots ,0\right)}^{T}$. The optimal such linear combination is that maximizing the noncentrality parameter

(2) $${\underline{c}}_{j}^{T}{\underline{\mu }}_{j}/{\left({\underline{c}}_{j}^{T}{𝒱}_{j}{\underline{c}}_{j}\right)}^{1/2},\;{\underline{\mu }}_{j}={\left(\mu_{1},\ldots ,\mu_{j},0,\ldots ,0\right)}^{T}.$$

Maximizing the noncentrality parameter (2) can be accomplished using the Cauchy‐Schwartz inequality, analogous to Li and Lagakos [13], leading to the optimal choice of ${\underline{c}}_{j}$ maximizing power given by ${\underline{c}}_{j}^{opt}\propto {𝒱}_{j}^{-}{\underline{\mu }}_{j}$. This result suggests sequentially computing the optimal statistics

(3) $$Y_{j,n}^{opt}\propto {\underline{\mu }}_{j}^{T}{𝒱}_{j}^{-}{\underline{X}}_{j,n}.$$

Letting b∝μ, Theorem 1 can be used to show that $Y_{n}^{opt}={\left(Y_{1,n}^{opt},\ldots ,Y_{K,n}^{opt}\right)}^{T}$ has the independent increments property.

Taking linear combinations in this way leads not only to statistics that yield powerful tests at each $t_{j}$ for detecting the alternative hypothesis $H_{A,n}$, but also results in test statistics that have the desired independent increments property under $H_{0}$; that is, $Y_{n}^{opt}={\left(Y_{1,n}^{opt},\ldots ,Y_{K,n}^{opt}\right)}^{T}\overset{\cdot }{˜}N(0,{𝒱}_{Y^{opt}})$, where ${𝒱}_{Y^{opt}}(j,j)={\underline{\mu }}_{j}^{T}{𝒱}_{j}^{-}{\underline{\mu }}_{j}$, and ${𝒱}_{Y^{opt}}(j,k)={𝒱}_{Y^{opt}}(j,j)$ for j≤k. The test at the jth interim analysis is based on the standardized test statistic

(4) $$Y_{j,n}^{opt}/{\left({\underline{\mu }}_{j}^{T}{𝒱}_{j}^{-}{\underline{\mu }}_{j}\right)}^{1/2}.$$

Because of the independent increments property, level α sequential tests and boundaries can be computed readily using standard methods and existing software. Note that, for standardized test statistics, dependence on n in the numerator and denominator cancels, so that all necessary calculations depend only on the data that have accumulated at each interim analysis.

The above rationale for choosing b to achieve high power at each interim analysis, given an alternative of interest, has intuitive appeal. In fact, it can be shown that choosing b in this way leads to the most powerful test that can be constructed for a specified level of significance α and α‐spending function, including relative to tests that are not based on linear combinations of $X_{1,n},\ldots ,X_{K,n}$. A proof is presented in the Supporting Information.

The foregoing development assumes that a specific type of alternative to the null hypothesis is of interest, and the test statistic (4) with b chosen proportional to μ is derived to have high power against that type of alternative. If in truth the distribution away from the null hypothesis is not consistent with such alternatives, then the test statistics derived above will not necessarily be optimal, but they will still have the independent increment property under $H_{0}$ because Theorem 1 guarantees that this property will hold for any arbitrary b. Thus, group sequential methods applied to them will preserve the type I error.

### 3.2 Considerations for Efficient Tests

If the original statistics $X_{1,n},\ldots ,X_{K,n}$ are indeed efficient for the alternative $H_{A,n}$, for example, as for a score test for treatment effect parameters in a relevant parametric or semiparametric model, then it is well known that $X_{n}$ has the independent increments property, so that the covariance matrix 𝒱 satisfies 𝒱(j,k)=𝒱(j,j), j≤k, and the mean under the alternative hypothesis is proportional to the variance; that is, $\mu_{j}=\gamma 𝒱(j,j)$. Then, from (3),

$$Y_{j,n}^{opt}=\{\gamma 𝒱(1,1),\ldots ,\gamma 𝒱(j,j),0,\ldots ,0\}{𝒱}_{j}^{-}{\underline{X}}_{j,n}.$$

However, under independent increments, the row vector {𝒱(1,1),…,𝒱(j,j),0,…,0} is the same as the jth row of ${𝒱}_{j}$. Thus,

$$\{\gamma 𝒱(1,1),\ldots ,\gamma 𝒱(j,j),0,\ldots ,0)\}{𝒱}_{j}^{-}=\gamma (0,\ldots ,0,1,0,\ldots ,0),$$

a row vector with jth element γ and zeros otherwise, so that $Y_{j,n}^{opt}=\gamma X_{j,n}$, j=1,…,K, and the standardized test statistic as in (4) based on $Y_{j,n}^{opt}$ is the same as the standardized test statistic based on $X_{j,n}$. That is, if we start with efficient tests, then the linear transformation for improvement leads us back to the same set of tests.

In some settings, the treatment effect can be characterized in terms of a scalar parameter θ in a parametric or semiparametric model, with null hypothesis $H_{0}:\theta =0$, and a consistent and asymptotically normal estimator for θ is available that can be used to form a test statistic for $H_{0}$. Let ${\hat{\theta }}_{j}$ be the estimator for θ based on all the available data through interim time $t_{j}$, j=1,…,K, and define $\underline{\hat{\vartheta }}={\left({\hat{\theta }}_{1},\ldots ,{\hat{\theta }}_{K}\right)}^{T}$. These estimators may or may not be efficient estimators for θ in the sense of achieving the Fisher information bound. First consider the case where the estimators are indeed efficient, under which Scharfstein et al. [5] and Jennison and Turnbull [6] have shown that $X_{n}=n^{1/2}\underline{\hat{\vartheta }}$ has the independent increments property under $H_{0}$. Importantly, in the case of sequentially‐computed estimators, with ${𝒱}_{\theta }$ the (asymptotic) covariance matrix of $X_{n}$, the independent increments property takes the form

(5) $${𝒱}_{\theta }(j,k)={𝒱}_{\theta }(k,k),\;\;\;\text{for}\;j\leq k,$$

where ${𝒱}_{\theta }(j,k)$ is the (j,k)th element of ${𝒱}_{\theta }$. Here, the variances ${𝒱}_{\theta }(j,j)$ of $X_{j,n}=n^{1/2}{\hat{\theta }}_{j}$, j=1,…,K, decrease with j as more data accrue, and the covariance between $n^{1/2}{\hat{\theta }}_{j}$ and $n^{1/2}{\hat{\theta }}_{k}$ is equal to the variance of the latter.

Under a local alternative $H_{A,n}:\theta =\theta_{n}$, where $n^{1/2}\theta_{n}\to \tau$, $X_{n}=n^{1/2}\underline{\hat{\vartheta }}$ converges in distribution to a normal random vector with mean $\tau 1_{K}$ and covariance matrix ${𝒱}_{\theta }$, where $1_{K}={\left(1,\ldots ,1\right)}^{T}$ is a K‐dimensional vector of ones. Thus, the optimal choice $b^{opt}$ for the vector b is proportional to $1_{K}$; and, defining ${𝒱}_{\theta ,j}$ and ${𝒱}_{\theta ,j}^{-}$ analogously to ${𝒱}_{j}$ and ${𝒱}_{j}^{-}$, respectively, the optimal choice for $Y_{j,n}^{opt}$ is given by

$$Y_{j,n}^{opt}=1_{j}^{T}{𝒱}_{\theta ,j}^{-}X_{j,n}=n^{1/2}1_{j}^{T}{𝒱}_{\theta ,j}^{-}{\underline{\hat{\vartheta }}}_{j},$$

where $1_{j}={\left(1,\ldots ,1,0,\ldots ,0\right)}^{T}$ is the K‐dimensional vector with j ones followed by (K−j) zeros, and ${\underline{\hat{\vartheta }}}_{j}={\left({\hat{\theta }}_{1},\ldots ,{\hat{\theta }}_{j},0,\ldots ,0\right)}^{T}$. Under the independent increments structure (5) for estimators, the jth row of ${𝒱}_{\theta ,j}$ is equal to ${𝒱}_{\theta ,j}(j,j)1_{j}^{T}$. Consequently, ${𝒱}_{\theta ,j}(j,j)1_{j}^{T}{𝒱}_{\theta ,j}^{-}=(0,\ldots ,0,1,0,\ldots ,0)$ defined above, so that $1_{j}^{T}{𝒱}_{\theta ,j}^{-}=(0,\ldots ,0,1/{𝒱}_{\theta ,j}(j,j),0,\ldots ,0)$, and

$$Y_{j,n}^{opt}=n^{1/2}{\hat{\theta }}_{j}/{𝒱}_{\theta ,j}(j,j).$$

That is, the optimal linear combination of ${\hat{\theta }}_{1},\ldots ,{\hat{\theta }}_{j}$ leading to $Y_{j,n}^{opt}$ involves only ${\hat{\theta }}_{j}$. Thus, for j≤k

$$\begin{array}{ll} \text{cov}(Y_{j,n}^{opt},Y_{k,n}^{opt}) & =n\;\text{cov}({\hat{\theta }}_{j},{\hat{\theta }}_{k})/\{{𝒱}_{\theta ,j}(j,j){𝒱}_{\theta ,k}(k,k)\}\\ & ={𝒱}_{\theta ,k}(k,k)/\{{𝒱}_{\theta ,j}(j,j){𝒱}_{\theta ,k}(k,k)\}\\ & =1/{𝒱}_{\theta ,j}(j,j)=\mathrm{var}(Y_{j,n}^{opt}), \end{array}$$

demonstrating independent increments. Moreover, the mean of $Y_{j,n}^{opt}$ is equal to $\tau /{𝒱}_{\theta ,j}(j,j)=\tau \mathrm{var}(Y_{j,n}^{opt})$, and it is straightforward that the standardized test statistic $Y_{j,n}^{opt}/{\left\{\mathrm{var}(Y_{j,n}^{opt})\right\}}^{1/2}$ is the same as the standardized test statistic $n^{1/2}{\hat{\theta }}_{j}/{\left\{{𝒱}_{\theta ,j}(j,j)\right\}}^{1/2}={\hat{\theta }}_{j}/{\left\{\mathrm{var}({\hat{\theta }}_{j})\right\}}^{1/2}$. That is, using efficient linear combinations of efficient estimators resulting in the independent increments property to construct a test statistic for $H_{0}:\theta =0$ leads to the same result as that using the efficient estimators alone. In practice, an estimator for $\mathrm{var}({\hat{\theta }}_{j})$ would be substituted.

In the case where the estimators for θ are not efficient and thus do not necessarily have the independent increments property, it is advantageous to take such linear combinations. Specifically, taking $b=1_{K}$, compute ${\left(Y_{1,n}^{opt},\ldots ,Y_{K,n}^{opt}\right)}^{T}$, where $Y_{j,n}^{opt}=n^{1/2}1_{j}^{T}{𝒱}_{\theta ,j}^{-}{\underline{\hat{\vartheta }}}_{j}$, and reject the null hypothesis whenever the standardized test statistic

$$n^{1/2}1_{j}^{T}{𝒱}_{\theta ,j}^{-}{\underline{\hat{\vartheta }}}_{j}/{\left\{1_{j}^{T}{𝒱}_{\theta ,j}^{-}1_{j}\right\}}^{1/2}$$

exceeds some boundary value. In practice, an estimator for ${𝒱}_{\theta ,j}$ would be substituted. By construction, these tests have the independent increments property, so that standard group sequential software can be used to compute the boundary values, and will have higher power than the usual test based on the statistic ${\hat{\theta }}_{j}/{\left\{\mathrm{var}({\hat{\theta }}_{j})\right\}}^{1/2}$. This approach is used by Van Lancker et al. [7] in the situation described in Section 1. Namely, by taking linear combinations of sequentially‐computed, potentially inefficient covariate adjusted estimators ${\hat{\theta }}_{j}$, j=1,…,K, that do not have the independent increments property, these authors derived a test enjoying this property that is potentially more efficient.

## 4 Examples

### 4.1 Preliminaries

We now consider two settings involving a time to event outcome where an existing test procedure for a relevant null hypothesis $H_{0}$ of no treatment effect is based on statistics without the independent increments property. For each, we demonstrate how the foregoing developments can be used to obtain modified statistics that have this property and target specific types of alternatives of interest. As is the case in many such formulations, the derivation is based on identifying the influence function [14] associated with the original test statistic under $H_{0}$ by representing the statistic as asymptotically equivalent to a sum of independent and identically distributed (iid) quantities, from which obtaining the covariance matrix of sequentially‐computed statistics is straightforward.

Consider a clinical trial comparing two treatments coded as 0 and 1, where up to n individuals enter in a staggered fashion at iid times $E_{1}\leq \cdots \leq E_{n}$ measured from the start of the trial at time t=0. For definiteness, suppose that interim analyses potentially are to be conducted at times $t_{1}<\cdots <t_{K}$, with $t_{1}>0$. Let Z∈{0,1} indicate randomized treatment assignment, and denote the event time for an arbitrary individual, measured from study entry, by T. Define the treatment‐specific time to event/survival distributions as $S_{z}(u)=P(T\geq u|Z=z)$, with corresponding hazard functions $\lambda_{z}(u)$, z=0,1. The event time T may be right censored, for example, due to loss to follow up. As is standard, letting C denote potential censoring time, U=min(T,C), Δ=I(T≤C) are observed, where I(·) is the indicator function, and we assume censoring is noninformative in the sense that T⊥C|Z, where “⊥” denotes “independent of.” Under these conditions, it is well known that the cause‐specific hazard functions $\lambda_{z}^{U}(u)=\lim_{du\to 0}{\left(du\right)}^{-1}P(u\leq U<u+du,\Delta =1|U\geq u,Z=z)$, which are identifiable from the data, are the same as $\lambda_{z}(u)$, z=0,1. Assuming that subjects enter the trial according to a completely random process, E⊥(U,Δ,Z), and, indexing subjects by i, we take $\left(E_{i},Z_{i},U_{i},\Delta_{i}\right)$, i=1,…,n, to be iid. At interim analysis time t, we observe data only for individuals i already enrolled in the trial, for whom $E_{i}\leq t$; and further administrative censoring is induced: for these individuals, we observe $\left(U_{i},\Delta_{i}\right)$ if $U_{i}\leq t-E_{i}$; otherwise, $U_{i}$ is censored at time $t-E_{i}$.

### 4.2 Interim Monitoring Based on Gehan's Wilcoxon Test

In the setting of time to event outcome, a null hypothesis of central interest is that of equality of the treatment‐specific survival distributions; that is, $H_{0}:S_{1}(u)=S_{0}(u)$ for all u≥0, which is equivalent to that of equality of the corresponding hazard functions, $H_{0}:\lambda_{1}(u)=\lambda_{0}(u)$. As in Section 1, when the assumption of proportional hazards is likely to be violated, analysts may wish to base a test of $H_{0}$ on a test statistic other than that for the standard logrank test. When differences in survival are anticipated to occur early in time, Gehan's Wilcoxon test, which may be more sensitive to such differences, is an attractive alternative; for example, Jiang et al. [15] demonstrate this advantage in analyses of data from the National Institute on Aging Interventions Testing Program, where the interventions compared are expected to influence mortality prior to middle age but not afterward.

Slud and Wei [12] have shown that the statistics on which Gehan's Wilcoxon test is based with censored survival data, computed sequentially, do not have the independent increments property, complicating the test's use in the context of interim monitoring. These authors demonstrate how group sequential tests that preserve the desired significance level can be constructed using an α‐spending function and recursively computing multivariate normal integrals to obtain stopping boundaries based on the asymptotic joint distribution of the statistics under the null hypothesis. However, given the appeal of using standard software for this purpose, we show how Theorem 1 can be used to obtain modified statistics that do have the independent increments property by identifying appropriate linear combinations of the sequentially‐computed statistics while not sacrificing power.

Under $H_{0}$, $S_{0}(u)=S_{1}(u)=S(u)$ and $\lambda_{1}(u)=\lambda_{0}(u)=\lambda (u)$, say. Define the event time counting process 𝒩(u)=I(U≤u,Δ=1) and at risk process 𝒴(u)=I(U≥u), and let dℳ(u)=d𝒩(u)−λ(u)𝒴(u)du denote the associated martingale increment. Tarone and Ware [16] have shown that Gehan's Wilcoxon test statistic with censored data can be written equivalently as a weighted logrank test; namely, at time t, the (normalized) statistic on which the test is based is given by

$$\begin{array}{ll} G_{n}(t) & =n^{-1⁄2}\sum_{i=1}^{n}I(E_{i}\leq t)\int_{0}^{t}\frac{W_{n}(u,t)}{n}\\ & \;\times \{Z_{i}-\bar{Z}(u,t)\}dN_{i}(u)I(t-E_{i}\geq u),\\ W_{n}(u,t) & =\sum_{i=1}^{n}I(U_{i}\geq u,t-E_{i}\geq u), \end{array}$$

and

$$\bar{Z}(u,t)=\frac{\sum_{i=1}^{n}Z_{i}I(U_{i}\geq u,t-E_{i}\geq u)}{\sum_{i=1}^{n}I(U_{i}\geq u,t-E_{i}\geq u)}.$$

By an algebraic identity, under $H_{0}$,

$$\begin{array}{ll} G_{n}(t) & =n^{-1⁄2}\sum_{i=1}^{n}I(E_{i}\leq t)\int_{0}^{t}\frac{W_{n}(u,t)}{n}\\ & \;\times \{Z_{i}-\bar{Z}(u,t)\}d{ℳ}_{i}(u)I(t-E_{i}\geq u), \end{array}$$

and it can be shown that

$$G_{n}(t)=n^{-1/2}\overset{n}{\underset{i=1}{\sum }}IF_{i}(t)+o_{P}(1),$$

where

(6) $$IF_{i}(t)=I(E_{i}\leq t)\int_{0}^{t}w(u,t)\{Z_{i}-\pi (u,t)\}\;d{ℳ}_{i}(u)I(t-E_{i}\geq u)$$

is the ith influence function of $G_{n}(t)$, $o_{P}(1)$ is a term that converges to zero as n→∞, and

$$\begin{array}{ll} n^{-1}W_{n}(u,t)\overset{p}{\to }w(u,t) & =P(U\geq u,t-E\geq u),\\ \bar{Z}(u,t)\overset{p}{\to }\pi (u,t) & =E\{ZI(U\geq u,t-E\geq u)\}\\ & \;÷E\{I(U\geq u,t-E\geq u)\}, \end{array}$$

where “$\overset{p}{\to }$” denotes convergence in probability. Thus,

$$G_{n}(t)\overset{D}{\to }N\left[0,\mathrm{var}\{IF(t)\}\right]\text{under}H_{0},$$

and $\left\{G_{n}(s),G_{n}(t)\right\}$, s<t, converges in distribution under $H_{0}$ to a bivariate normal random vector with mean zero and covariance matrix

$$\left[\begin{array}{cc} \mathrm{var}\{IF(s)\} & \text{cov}\{IF(s),IF(t)\}\\ \text{cov}\{IF(s),IF(t)\} & \mathrm{var}\{IF(t)\} \end{array}\right].$$

We show in the Supporting Information that

(7) $$\mathrm{var}\{IF(t)\}=\int_{0}^{t}w^{3}(u,t)\pi (u,t)\{1-\pi (u,t)\}\lambda (u)du,$$

and, for s≤t,

(8) $$\text{cov}\{IF(s),IF(t)\}=\int_{0}^{s}w^{2}(u,s)w(u,t)\pi (u,s)\{1-\pi (u,s)\}\lambda (u)du.$$

From (7) and (8), in general, cov{IF(s),IF(t)}≠var{IF(s)} for s≤t, and thus the statistic $G_{n}(t)$ does not have the independent increments property.

Let $X_{j,n}=G_{n}(t_{j})$, j=1,…,K, denote the original sequential statistics, and let ${𝒱}_{IF}$ be the (K×K) asymptotic covariance matrix of ${\left(X_{1,n},\ldots ,X_{K,n}\right)}^{T}$, obtained from (7) and (8). Gehan's Wilcoxon test statistic at the jth interim analysis would be computed as

$$X_{j,n}/{\left\{{\hat{𝒱}}_{IF}(j,j)\right\}}^{1/2},$$

where ${\hat{𝒱}}_{IF}$ is an estimator for ${𝒱}_{IF}$ obtained by estimating (7) and (8). Namely, using standard counting process methods, var{IF(t)} in (7) can be estimated by

(9) $$\begin{array}{ll} \hat{\mathrm{var}}\{IF(t)\} & =\int_{0}^{t}{\left\{\frac{W_{n}(u,t)}{n}\right\}}^{3}\bar{Z}(u,t)\{1-\bar{Z}(u,t)\}\frac{dN(u,t)}{W_{n}(u,t)}\\ & =\int_{0}^{t}\left\{\frac{W_{n}{\left(u,t\right)}^{2}}{n^{3}}\right\}\bar{Z}(u,t)\{1-\bar{Z}(u,t)\}dN(u,t), \end{array}$$

where $N(u,t)=\sum_{i=1}^{n}I(U_{i}\leq u,\Delta_{i}=1,U_{i}\leq t-E_{i})$, and, similarly, a consistent estimator for cov{IF(s),IF(t)} in (8) is given by

(10) $$\begin{array}{ll} \hat{\text{cov}}\{IF(s),IF(t)\} & =\int_{0}^{s}\left\{\frac{W_{n}(u,s)W_{n}(u,t)}{n^{3}}\right\}\\ & \;\times \bar{Z}(u,s)\{1-\bar{Z}(u,s)\}dN(u,s). \end{array}$$

We now describe several approaches to choosing linear combinations of the sequentially‐computed statistics $X_{j,n}$ that result in statistics that do have the independent increments property, which involve choice of the vector of constants $b={\left(b_{1},\ldots ,b_{K}\right)}^{T}$. In the first two approaches, we choose b to target specific alternative hypotheses as in Section 3.1. It is well known that the Wilcoxon test in the case of no censoring has high power to detect log‐odds alternatives; that is, where

(11) $$\begin{array}{ll} \log\left\{\frac{S_{1}(u)}{1-S_{1}(u)}\right\} & =\log\left\{\frac{S_{0}(u)}{1-S_{0}(u)}\right\}+\delta ,\text{equivalently}\\ S_{1}(u) & =S_{1}(u,\delta )=\frac{S_{0}(u)\exp(\delta )}{1+S_{0}(u)\{\exp(\delta )-1\}}, \end{array}$$

and δ denotes the treatment effect parameter. We show in the Supporting Information that, under local log‐odds alternatives with treatment effect parameters $\delta_{n}$ such that $n^{1/2}\delta_{n}\to \tau$, the resulting Gehan's Wilcoxon statistic $G_{n}(t)$ converges in distribution to a normal random variable with variance var{IF(t)} (same as under the null hypothesis) but with mean

(12) $$\mu (t)=-\tau \int_{0}^{t}w^{2}(u,t)\pi (u,t)\{1-\pi (u,t)\}S(u)\lambda (u)du.$$

A consistent estimator for μ(t) in (12) is given by

(13) $$\hat{\mu }(t)=-\tau \int_{0}^{t}\left\{\frac{W_{n}(u,t)}{n^{2}}\right\}\bar{Z}(u,t)\{1-\bar{Z}(u,t)\}\hat{S}(u,t)dN(u,t),$$

where $\hat{S}(u,t)$ is the Kaplan–Meier estimator of the survival distribution under $H_{0}$ using all the available censored survival data combined over both treatments through time t. The proposed test statistic at the jth interim analysis is then

(14) $$Y_{j,n}/{\left({\underline{\hat{\mu }}}_{j}^{T}{\hat{𝒱}}_{IF,j}^{-}{\underline{\hat{\mu }}}_{j}\right)}^{1/2},\;Y_{j,n}={\underline{\hat{\mu }}}_{j}^{T}{\hat{𝒱}}_{IF,j}^{-}{\underline{X}}_{j,n},$$

where, with $\hat{\mu }(t)$ as in (13), ${\underline{\hat{\mu }}}_{j}={\left\{\hat{\mu }(t_{1}),\ldots ,\hat{\mu }(t_{j}),0,\ldots ,0\right\}}^{T}$, and ${\hat{𝒱}}_{IF,j}$ is the (K×K) matrix with upper left hand (j×j) submatrix that of ${\hat{𝒱}}_{IF}$ and the remainder of the matrix zeros. The $Y_{j,n}$, j=1,…,K, have the independent increments property, and the test should be more powerful against the log‐odds alternative than that based on the original Wilcoxon statistics $X_{1,n},\ldots ,X_{j,n}$.

In some settings, a treatment may be expected to have a delayed effect. We thus consider the alternative hypothesis where

(15) $$\lambda_{1}(u)=\lambda_{1}(u,\delta )=\lambda_{0}(u)I(u\leq {𝒯}_{delay})+\lambda_{0}(u)\exp(\delta )I(u>{𝒯}_{delay});$$

that is, the hazard function for treatment 1 is the same as that for treatment 0 through some delay time ${𝒯}_{delay}$ and then is proportional to that for treatment 0 by a proportionality constant exp(δ). We refer to this as a non‐proportional hazards alternative, with the null hypothesis being δ=0. We show in the Supporting Information that, under local alternatives $\delta_{n}$ such that $n^{1/2}\delta_{n}\to \tau$, the Gehan's Wilcoxon statistic $G_{n}(t)$ converges in distribution to a normal random variable with variance var{IF(t)}, but mean

(16) $$\mu (t)=-\tau \int_{{𝒯}_{delay}}^{t}w^{2}(u,t)\pi (u,t)\{1-\pi (u,t)\}\lambda (u)du.$$

A consistent estimator for μ(t) in (16) is given by

(17) $$\hat{\mu }(t)=-\tau \int_{{𝒯}_{delay}}^{t}\left\{\frac{W_{n}(u,t)}{n^{2}}\right\}\bar{Z}(u,t)\{1-\bar{Z}(u,t)\}dN(u,t).$$

As above, the test statistic at the jth interim analysis is of the form (14), where now ${\underline{\hat{\mu }}}_{j}={\left\{\hat{\mu }(t_{1}),\ldots ,\hat{\mu }(t_{j}),0,\ldots ,0\right\}}^{T}$ with $\hat{\mu }(t)$ as in (17), and the $Y_{j,n}$, j=1,…,K, have the independent increments property, so that the test should be more powerful against this alternative than that based on $X_{1,n},\ldots ,X_{j,n}$. Note that if ${𝒯}_{delay}=0$, then (15) reduces to a proportional hazards alternative.

An ad hoc approach to choosing b without specifying a targeted alternative is to proceed as if the statistics were efficient with independent increments, in which case the mean of the statistics would be (asymptotically) proportional to the variance; that is, $\mu_{j}=E(X_{j,n})=E\{G_{n}(t_{j})\}\propto \mathrm{var}\{G_{n}(t_{j})\}=\mathrm{var}\{IF(t_{j})\}$, j=1,…,K. Then, as above, b would be chosen to be proportional to ${\left[\mathrm{var}\{IF(t_{1})\},\ldots ,\mathrm{var}\{IF(t_{K})\}\right]}^{T}$. Although the Wilcoxon statistics are not efficient, this approach is simple to implement and leads to the modified statistics

$$Y_{j,n}={\hat{\underline{b}}}_{j}^{T}{\hat{𝒱}}_{IF,j}^{-}{\underline{X}}_{j,n},$$

where ${\hat{\underline{b}}}_{j}={\left[\hat{\mathrm{var}}\{IF(t_{1})\},\ldots ,\hat{\mathrm{var}}\{IF(t_{j})\},0,\ldots ,0\right]}^{T}$, which have the independent increments property.

We demonstrate the performance of these tests in a suite of simulation studies, each involving 10 000 Monte Carlo group sequential trials. In all studies, $S_{0}(u)$ follows an exponential distribution with constant hazard rate equal to 1, $S_{0}(u)=\exp(-u)$. We consider three sets of alternatives: proportional hazards, where $S_{1}(u)=S_{1}(u,\delta )$ is exponential with constant hazard rate exp(δ), $S_{1}(u)=S_{1}(u,\delta )=\exp\{-u\exp(\delta )\}$; log‐odds as in (11); and non‐proportional hazards as in (15) with ${𝒯}_{delay}=0.5$. In all three cases, δ=0 corresponds to the null hypothesis $H_{0}$. In each trial, individuals enter according to a uniform U(0,2) distribution and are randomized to treatments 0 and 1 with equal probability, π=P(Z=1)=P(Z=0)=0.5. For each individual, with Z=z, T has distribution corresponding to $S_{z}(u)$, z=0,1, C follows an exponential distribution with hazard rate equal to 0.25, and U=min(T,C) and Δ=I(T≤C). Each trial is monitored at K=5 interim analysis times t=(1.5,1.75,2.0,2.5,3.0), and in all cases the nominal significance level α=0.05.

We consider several group sequential test procedures based on Gehan's Wilcoxon test. The first, denoted as the unadjusted Wilcoxon test, does not take account of the fact that the usual Gehan's Wilcoxon statistics do not have the independent increments property and naively computes stopping boundaries using standard methods for tests based on independent increments. The second, referred to as the adjusted Wilcoxon test, is based on the usual Gehan's Wilcoxon statistics, with boundaries computed to preserve the desired significance level using an α‐spending function and recursive multivariate normal integration using the mvtnorm package in R [17] based on the asymptotic joint distribution of the sequentially‐computed statistics under the null hypothesis, as proposed by Slud and Wei [12]. Four additional tests based on the modified Wilcoxon statistics leading to independent increments are also evaluated: the ad hoc version with the constant vector b chosen to be proportional to the variance of the sequentially‐computed Wilcoxon statistic, denoted Wilcoxon I; that with b chosen to favor log‐odds alternatives according to (13), Wilcoxon II; that with b chosen to favor proportional hazards alternatives according to (17) with ${𝒯}_{delay}=0$, Wilcoxon III; and that with b chosen to favor non‐proportional hazards alternatives according to (17) with ${𝒯}_{delay}=0.5$, Wilcoxon IV. For comparison, we consider the logrank test, which has power targeted for proportional hazards alternatives.

In each study, the potential sample size n=1000, and we use an α‐spending function at each of the five interim analysis times of (0.05,0.1,0.4,0.7,1)×0.05. All tests are two‐sided, and $H_{0}$ is rejected whenever the absolute value of the standardized test statistics exceeds the boundary values, which for the unadjusted Wilcoxon and Wilcoxon I‐IV are computed in all cases using the ldbounds package in R [18] and for the adjusted Wilcoxon are computed as above. For each simulation scenario, we compute the empirical level and power of each test as the proportion of the 10 000 trials for which the null hypothesis is rejected and also report the Monte Carlo average of the number of analyses conducted before stopping the trial.

Results are presented in Table 1. Under the null hypothesis, all tests except the unadjusted Wilcoxon have empirical type I error close to the nominal 0.05, which is not surprising given that incorrect stopping boundaries are used for the latter. As expected, the logrank test has greatest power to detect a proportional hazards alternative, followed by the Wilcoxon III, which targets this alternative. The Wilcoxon II test, which targets log‐odds alternatives, has the highest power in this scenario, along with the adjusted Wilcoxon test. The greatest difference in power across tests occurs under the non‐proportional hazards alternative, where the Wilcoxon IV test targeting such alternatives and the logrank test achieve considerably higher power than the other tests. The adjusted Wilcoxon test achieves similar power to Wilcoxon II across alternatives. The average number of analyses conducted is similar across tests but somewhat lower for those with higher power, as expected.

**TABLE 1:** Probability of rejecting the null hypothesis and average number of analyses before stopping based on 10 000 Monte Carlo group sequential trials.

Test^a^	Null	Prop‐haz	Log‐odds	Non‐prop‐haz
	δ=0.00	δ=0.23	δ=0.32	δ=0.47
Wilcoxon (unadjusted)	0.044	0.745 (3.62)^b^	0.779 (3.29)	0.252 (4.83)
Wilcoxon (adjusted)	0.050	0.763 (3.56)	0.796 (3.23)	0.270 (4.81)
Wilcoxon I	0.049	0.740 (3.64)	0.744 (3.34)	0.372 (4.76)
Wilcoxon II	0.050	0.773 (3.56)	0.796 (3.23)	0.316 (4.80)
Wilcoxon III	0.049	0.787 (3.55)	0.786 (3.23)	0.489 (4.78)
Wilcoxon IV	0.048	0.677 (3.72)	0.658 (3.43)	0.736 (4.71)
Logrank	0.048	0.840 (3.25)	0.771 (3.29)	0.749 (4.17)
RMST	0.047	0.833 (3.27)	0.707 (3.43)	0.822 (3.87)
RMST I	0.050	0.824 (3.28)	0.732 (3.39)	0.736 (3.99)
RMST II	0.049	0.835 (3.26)	0.720 (3.41)	0.806 (3.90)
RMST III	0.050	0.802 (3.36)	0.651 (3.54)	0.858 (3.79)

Abbreviations: Null, null hypothesis; Prop‐haz, proportional hazards alternative; Log‐odds, log odds alternative; Non‐prop‐haz, non‐proportional hazards alternative.

^a^ Tests are as defined in the text.

^b^ Monte Carlo average of number of interim analyses before stopping in parentheses.

Table 2 shows the Monte Carlo average of the standardized covariance matrix of the statistics used in constructing each test under $H_{0}$, obtained by dividing the empirical covariance matrix by the empirical variance of $X_{K,n}$. As expected, the statistics associated with the logrank test and the Wilcoxon I, II, III, and IV tests all have empirical standardized covariance matrices consistent with independent increments; that associated with the adjusted Wilcoxon (usual Gehan's Wilcoxon) test does not.

**TABLE 2:** Monte Carlo average empirical standardized covariance matrix for each test under the null hypothesis.

Wilcoxon (adjusted)						Wilcoxon I						Wilcoxon II				
0.271	0.321	0.371	0.405	0.408		0.231	0.228	0.227	0.226	0.227		0.591	0.587	0.586	0.586	0.587
0.321	0.450	0.521	0.572	0.575		0.228	0.431	0.429	0.427	0.427		0.587	0.708	0.707	0.706	0.706
0.371	0.521	0.701	0.771	0.776		0.227	0.429	0.711	0.709	0.708		0.586	0.707	0.831	0.830	0.829
0.405	0.572	0.771	0.962	0.972		0.226	0.427	0.709	0.976	0.973		0.586	0.706	0.830	0.972	0.970
0.408	0.575	0.776	0.972	1.000		0.227	0.427	0.708	0.973	1.000		0.587	0.706	0.829	0.970	1.000

Wilcoxon III						Wilcoxon IV						Logrank				
0.432	0.429	0.428	0.428	0.429		0.017	0.017	0.017	0.017	0.017		0.478	0.474	0.470	0.472	0.471
0.429	0.533	0.532	0.531	0.531		0.017	0.038	0.037	0.037	0.037		0.474	0.595	0.589	0.591	0.589
0.428	0.532	0.641	0.640	0.639		0.017	0.037	0.061	0.061	0.060		0.470	0.589	0.711	0.712	0.709
0.428	0.531	0.640	0.859	0.854		0.017	0.037	0.061	0.302	0.291		0.472	0.591	0.712	0.908	0.902
0.429	0.531	0.639	0.854	1.000		0.017	0.037	0.060	0.291	1.000		0.471	0.589	0.709	0.902	1.000

RMST						RMST I						RMST II				
0.527	0.502	0.463	0.438	0.445		0.652	0.649	0.644	0.648	0.647		0.468	0.465	0.459	0.463	0.462
0.502	0.629	0.587	0.550	0.551		0.649	0.755	0.747	0.750	0.750		0.465	0.588	0.579	0.582	0.580
0.463	0.587	0.699	0.655	0.648		0.644	0.747	0.839	0.841	0.840		0.459	0.579	0.699	0.699	0.697
0.438	0.550	0.655	0.864	0.843		0.648	0.750	0.841	0.966	0.963		0.463	0.582	0.699	0.899	0.891
0.445	0.551	0.648	0.843	1.000		0.647	0.750	0.840	0.963	1.000		0.462	0.580	0.697	0.891	1.000

RMST III				
0.152	0.149	0.145	0.148	0.147
0.149	0.301	0.293	0.294	0.292
0.145	0.293	0.449	0.447	0.443
0.148	0.294	0.447	0.761	0.747
0.147	0.292	0.443	0.747	1.000

### 4.3 Monitoring Using Restricted Mean Survival Time

Restricted mean survival time (RMST) is defined as R(L)=E{min(T,L)}, for some specified time L, mean survival time restricted to time L, which can also be computed as $R(L)=\int_{0}^{L}S(u)\;du$, the area under the survival distribution S(u) of T through time L. In many studies, because of limited follow up, it is not possible to estimate E(T) unless the support of T is contained in the interval from 0 to the maximum follow up time. Thus, of necessity, survival time must be restricted; for example, at an interim analysis at time t, survival time can be observed only through t. Given possibly censored observations on T, R(L) can be estimated nonparametrically by $\int_{0}^{L}\hat{S}(u)\;du$, where $\hat{S}(u)$ is the Kaplan–Meier estimator of S(u) based on these data. Consequently, the null hypothesis of equality of treatment‐specific survival distributions can be characterized in terms of the treatment effect parameter $\theta =R_{1}(L)-R_{0}(L)=\int_{0}^{L}\{S_{1}(u)-S_{0}(u)\}du$, the difference in treatment‐specific RMST, as $H_{0}:\theta =0$. This parameter has intuitive appeal, as it can be viewed also as representing the expected years of life saved by using the better treatment over the restricted time interval (0,L). Moreover, θ can be estimated nonparametrically by

$$\hat{\theta }=\int_{0}^{L}\{{\hat{S}}_{1}(u)-{\hat{S}}_{0}(u)\}du,$$

where ${\hat{S}}_{z}(u)$, z=0,1, are the treatment‐specific Kaplan–Meier estimators of $S_{z}(u)$, z=0,1.

Group sequential testing based on RMST has been discussed by several authors [9, 10]. At interim analysis times $t_{1},\ldots ,t_{K}$, one considers $\underline{\vartheta }={\left(\theta_{1},\ldots ,\theta_{K}\right)}^{T}$, where

(18) $$\begin{array}{ll} \theta_{j} & =R_{1}(L_{j})-R_{0}(L_{j})=\int_{0}^{L_{j}}\{S_{1}(u)-S_{0}(u)\}du,\\ j & =1,\ldots ,K,L_{j}\leq t_{j}. \end{array}$$

Because of limited follow up at $t_{j}$, the restricted time $L_{j}$ must be less than $t_{j}$, and $L_{j}$, j=1,…,K, may be taken to increase with increasing $t_{j}$ to reflect that additional data are accrued as the study progresses. The null hypothesis is then $H_{0}:\theta_{1}=\cdots =\theta_{K}=0$, and monitoring the study and testing the null hypothesis entails estimating $\underline{\vartheta }$ by some estimator $\underline{\hat{\vartheta }}={\left({\hat{\theta }}_{1},\ldots ,{\hat{\theta }}_{K}\right)}^{T}$ and rejecting $H_{0}$ at the first time the estimated treatment effect parameter (or absolute value thereof), suitably normalized and standardized, exceeds some critical value. Letting ${\hat{S}}_{z}(u,t)$ denote the Kaplan–Meier estimator for $S_{z}(u)$, z=0,1, based on all available data through time t, and defining ${\hat{R}}_{z}(t,L)=\int_{0}^{L}{\hat{S}}_{z}(u,t)\;du$, z=0,1, the obvious estimator for $\theta_{j}$ in (18) is

(19) $$\begin{array}{ll} {\hat{\theta }}_{j} & ={\hat{R}}_{1}(t_{j},L_{j})-{\hat{R}}_{0}(t_{j},L_{j})=\int_{0}^{L_{j}}\{{\hat{S}}_{1}(u,t_{j})-{\hat{S}}_{0}(u,t_{j})\}du,\\ j & =1,\ldots ,K. \end{array}$$

Taking $L_{j}$ not to vary with $t_{j}$, so that $L_{j}=L$ for all j=1,…,K, implies that monitoring does not begin until after L, that is, $t_{1}>L$, and that $\theta_{j}=\theta$, j=1,…,K. As demonstrated by Murray and Tsiatis [9], under these conditions, at each interim analysis, the common θ is estimated using the efficient Kaplan–Meier estimators for $S_{z}(u)$, z=0,1, and the sequentially‐computed estimators $\int_{0}^{L}\{{\hat{S}}_{1}(u,t_{j})-{\hat{S}}_{0}(u,t_{j})\}du$, suitably normalized, have the independent increments property, so that group sequential methods can be implemented readily with standard software. However, if $L_{j}$ and thus $\theta_{j}$ do vary over time as above, then normalized versions of ${\hat{\theta }}_{j}$ in (19) do not have this property. In this case, Murray and Tsiatis [9] and Lu and Tian [10] show that group sequential tests can be constructed that preserve the desired significance level by using an α‐spending function and recursively evaluating multivariate normal integrals to construct stopping boundaries based on the asymptotic joint distribution of suitably normalized ${\hat{\theta }}_{j}$ under the null hypothesis. As in Section 4.2, to take advantage of standard software for computing stopping boundaries, we now describe how Theorem 1 can be used to derive modified statistics based on linear combinations of sequentially‐computed estimators that have the independent increments property.

Let $n_{z}=\sum_{i=1}^{n}I(Z_{i}=z)$ denote the potential number of individuals randomized to treatment z, z=0,1, with entry times $E_{z,i}$ and event/censoring times and indicators $\left(U_{z,i},\Delta_{z,i}\right)$, $i=1,\ldots ,n_{z}$. Then $n=n_{0}+n_{1}$ is the total potential sample size, and, $n_{1}/n\to \pi =P(Z=1)$ as n→∞. We proceed analogous to the previous section and derive the asymptotic covariance matrix ${𝒱}_{\theta }$ of $n^{1/2}\hat{\underline{\vartheta }}=n^{1/2}{\left({\hat{\theta }}_{1},\ldots ,{\hat{\theta }}_{K}\right)}^{T}$. Following previous authors [9, 10] and owing to the form ${\hat{\theta }}_{j}$ in (19) as the difference of treatment‐specific terms, we first find the influence functions for ${\hat{R}}_{z}(t,L)$, z=0,1. The ith influence function $IF_{R,z,i}(t,L)$ for ${\hat{R}}_{z}(t,L)$ satisfies

$$n_{z}^{1/2}\{{\hat{R}}_{z}(t,L)-R_{z}(L)\}=n_{z}^{-1/2}\overset{n_{z}}{\underset{i=1}{\sum }}IF_{R,z,i}(t,L)+o_{P}(1).$$

We show in the Supporting Information that $IF_{R,z,i}(t,L)$ is given by

(20) $$IF_{R,z,i}(t,L)=\int_{0}^{L}\frac{A_{z}(u,L)}{w_{z}(u,t)}d{ℳ}_{z,i}(u)I(t-E_{z,i}\geq u),$$

where $A_{z}(u,L)=\int_{u}^{L}S_{z}(x)\;dx$. Accordingly, at $t_{j}$,

(21) $$n_{z}^{1/2}\{{\hat{R}}_{z}(t_{j},L_{j})-R_{z}(L_{j})\}\overset{D}{\to }N[\;0,\mathrm{var}\{IF_{R,z}(t_{j},L_{j})\}\;],$$

where (see the Supporting Information)

(22) $$\mathrm{var}\{IF_{R,z}(t_{j},L_{j})\}=\left\{\int_{0}^{L_{j}}\frac{A_{z}^{2}(u,L_{j})}{w_{z}(u,t_{j})}\lambda_{z}(u)du\right\}.$$

A consistent estimator for $\mathrm{var}\{IF_{R,z}(t_{j},L_{j})\}$ in (22) is given by, analogous to Nemes et al. [19],

(23) $$\begin{array}{ll} & n_{z}\int_{0}^{L_{j}}\frac{{\hat{A}}_{z}^{2}(u,t_{j},L_{j})}{W_{z,n}(u,t_{j})\{W_{z,n}(u,t_{j})-1\}}\;dN_{z}(u,t_{j}),\\ & \;{\hat{A}}_{z}(u,t,L)=\int_{u}^{L}{\hat{S}}_{z}(x,t)\;dx, \end{array}$$

where

$$\begin{array}{ll} W_{z,n}(u,t) & =\overset{n_{z}}{\underset{i=1}{\sum }}I(U_{z,i}\geq u,t-E_{z,i}\geq u),\\ N_{z}(u,t) & =\overset{n_{z}}{\underset{i=1}{\sum }}I(U_{z,i}\leq u,\Delta_{z,i}=1,U_{z,i}\leq t-E_{z,i}). \end{array}$$

Similarly, ${\left[\;n_{z}^{1/2}\{{\hat{R}}_{z}(t_{j},L_{j})-R_{z}(L_{j})\},n_{z}^{1/2}\{{\hat{R}}_{z}(t_{k},L_{k})-R_{z}(L_{k})\}\;\right]}^{T}$,j≤k converges in distribution to a bivariate normal random vector with mean zero and covariance matrix with diagonal elements $\mathrm{var}\{IF_{R,z}(t_{j},L_{j})\}$ and $\mathrm{var}\{IF_{R,z}(t_{k},L_{k})\}$ and off‐diagonal elements $\text{cov}\{IF_{R,z}(t_{j},L_{j}),IF_{R,z}(t_{k},L_{k})\}$. We show in the Supporting Information that

(24) $$\begin{array}{ll} & \text{cov}\{IF_{R,z,i}(t_{j},L_{j}),IF_{R,z,i}(t_{k},L_{k})\}\\ & \;=\int_{0}^{L_{j}}\frac{A_{z}(u,L_{j})A_{z}(u,L_{k})}{w_{z}(u,t_{k})}\lambda_{z}(u)\;du. \end{array}$$

Inspection of (22) and (24) shows that the independent increments property (for estimators) does not hold unless $L_{k}=L_{j}$. A consistent estimator for (24) is given by

(25) $$n_{z}\int_{0}^{L_{j}}\frac{{\hat{A}}_{z}(u,t_{k},L_{j}){\hat{A}}_{z}(u,t_{k},L_{k})}{W_{z,n}(u,t_{k})\{W_{z,n}(u,t_{k})-1\}}dN_{z}(u,t_{k}).$$

Note that in (25) we estimate both $A_{z}(u,L_{j})$ and $A_{z}(u,L_{k})$ using all the data through the larger time $t_{k}$.

From these results, the asymptotic distribution of $n^{1/2}\underline{\hat{\vartheta }}$ under $H_{0}$ and thus the form of ${𝒱}_{\theta }$ can be deduced; it suffices to derive the bivariate distribution of $n^{1/2}{\left({\hat{\theta }}_{j},{\hat{\theta }}_{k}\right)}^{T}$. Note that, under $H_{0}$, $R_{1}(L_{j})=R_{0}(L_{j})$, so that

$$\begin{array}{ll} & n^{1/2}{\hat{\theta }}_{j}\\ & \;=n^{1/2}\{{\hat{R}}_{1}(t_{j},L_{j})-{\hat{R}}_{0}(t_{j},L_{j})\}={\left(n/n_{1}\right)}^{1/2}n_{1}^{1/2}\{{\hat{R}}_{1}(t_{j},L_{j})\\ & \;-R_{1}(L_{j})\}-{\left(n/n_{0}\right)}^{1/2}n_{0}^{1/2}\{{\hat{R}}_{0}(t_{j},L_{j})-R_{0}(L_{j})\}\\ & \;\overset{D}{\to }N\left[0,\pi^{-1}\mathrm{var}\{IF_{R,1}(t_{j},L_{j})\}+{\left(1-\pi \right)}^{-1}\mathrm{var}\{IF_{R,0}(t_{j},L_{j})\}\right], \end{array}$$

using (21), Slutsky's theorem, and the fact that the treatment‐specific RMST estimators are from independent samples, and similarly for $n^{1/2}{\hat{\theta }}_{k}$. Moreover, using (24),

$$\begin{array}{ll} \text{cov}(n^{1/2}{\hat{\theta }}_{j},n^{1/2}{\hat{\theta }}_{k}) & =\pi^{-1}\int_{0}^{L_{j}}\frac{A_{1}(u,L_{j})A_{1}(u,L_{k})}{w_{1}(u,t_{k})}\lambda_{1}(u)\;du\\ & \;+{\left(1-\pi \right)}^{-1}\int_{0}^{L_{j}}\frac{A_{0}(u,L_{j})A_{0}(u,L_{k})}{w_{0}(u,t_{k})}\lambda_{0}(u)\;du. \end{array}$$

Thus, $n^{1/2}{\left({\hat{\theta }}_{j},{\hat{\theta }}_{k}\right)}^{T}$ converges in distribution to a normal random vector with mean zero and covariance matrix that follows from these results. An estimator ${\hat{𝒱}}_{\theta }$ for ${𝒱}_{\theta }$ follows by substitution of (23) and (25) in the foregoing expressions along with the estimator $\hat{\pi }=n_{1}/n$. With these substitutions, the standardized test statistic $n^{1/2}{\hat{\theta }}_{j}/{\left\{{\hat{𝒱}}_{\theta }(j,j)\right\}}^{1/2}$ does not depend n, $n_{1}$, or $n_{0}$ and thus depends only on the data accrued through $t_{j}$.

We are now in a position to define modified test statistics obtained by choosing linear combinations of the normalized, sequentially‐computed estimators that result in statistics with the independent increments property. We thus must choose the vector of constants $b={\left(b_{1},\ldots ,b_{K}\right)}^{T}$ accordingly. Let $X_{j,n}=n^{1/2}{\hat{\theta }}_{j}$ denote the original, sequentially‐computed statistics. Following Section 3.1, we demonstrate how to choose b to target the same specific alternatives as in Section 4.2. For a given such alternative $S_{1}(t,\delta )$ as in (11) and (15), under local alternatives $\delta_{n}$, such that $n^{1/2}\delta_{n}\to \tau$, $X_{j,n}$ converges in distribution to a normal random variable with variance ${𝒱}_{\theta }(j,j)$ and mean given by the limit of $n^{1/2}\int_{0}^{L_{j}}\{S_{1}(u,\delta_{n})-S_{0}(u)\}du$, which equals

$$\mu (L_{j})=\tau \int_{0}^{L_{j}}{\dot{S}}_{1}(u)\;du,\;{\dot{S}}_{1}(u)=\frac{dS_{1}(u,\delta )}{d\delta }{|}_{\delta =0}.$$

For the log‐odds alternative (11), ${\dot{S}}_{1}(u)=S_{0}(u)\{1-S_{0}(u)\}$, and the asymptotic mean can be estimated by

(26) $$\hat{\mu }(t_{j},L_{j})=\int_{0}^{L_{j}}\hat{S}(u,t_{j})\{1-\hat{S}(u,t_{j})\}du,$$

where as before $\hat{S}(u,t)$ is the Kaplan–Meier estimator of the survival distribution under $H_{0}$ using the data through time t combined over both treatments. For the non‐proportional hazards alternative (15), $S_{1}(u,\delta )=\exp\{-\Lambda_{1}(u,\delta )\}$, where $\Lambda_{1}(u,\delta )$ is the cumulative hazard function

$$\begin{array}{ll} \Lambda_{1}(u,\delta ) & =\int_{0}^{u}\lambda_{1}(x,\delta )\;dx=\Lambda_{0}(u)I(u<{𝒯}_{delay})\\ & \;+\{\Lambda_{0}(u)-\Lambda_{0}({𝒯}_{delay})\}\exp(\delta )I(u\geq {𝒯}_{delay}), \end{array}$$

and thus ${\dot{S}}_{1}(u)=-S_{0}(u)\{\Lambda_{0}(u)-\Lambda_{0}({𝒯}_{delay})\}I(u\geq {𝒯}_{delay})$, and the asymptotic mean can be estimated by

(27) $$\hat{\mu }(t_{j},L_{j})=\int_{{𝒯}_{delay}}^{L_{j}}\hat{S}(u,t_{j})\{\hat{\Lambda }(u,t_{j})-\hat{\Lambda }({𝒯}_{delay},t_{j})\}\;du,$$

where $\hat{\Lambda }(u,t)$ is the Nelson‐Aalen estimator for the cumulative hazard function under $H_{0}$ using the data through time t combined over both treatments. Proportional hazards alternatives can be targeted by taking ${𝒯}_{delay}=0$. As before, for the alternative of interest, the proposed test statistic at the jth interim analysis is

(28) $$Y_{j,n}/{\left({\underline{\hat{\mu }}}_{j}^{T}{\hat{𝒱}}_{\theta ,j}^{-}{\underline{\hat{\mu }}}_{j}\right)}^{1/2},\;Y_{j,n}={\underline{\hat{\mu }}}_{j}^{T}{\hat{𝒱}}_{\theta ,j}^{-}{\underline{X}}_{j,n},$$

where ${\underline{\hat{\mu }}}_{j}={\left\{\hat{\mu }(t_{1},L_{1}),\ldots ,\hat{\mu }(t_{j},L_{j}),0,\ldots ,0\right\}}^{T}$, and ${\hat{𝒱}}_{\theta ,j}$ is the (K×K) matrix where the upper left hand (j×j) submatrix is that of ${\hat{𝒱}}_{\theta }$ and the remainder of the matrix zeros. The $Y_{j,n}$, j=1,…,K, have the independent increments property, and the test should be more powerful against the targeted alternative than that based on $X_{1,n},\ldots ,X_{j,n}$.

We study the performance of group sequential test procedures based on RMST and compare to that of the procedures based on the Gehan's Wilcoxon and logrank tests in simulations studies involving the same 10 000 Monte Carlo trial data sets with n=1000 for each of the generative scenarios in Section 4.2, with significance level α=0.05 and the same five interim analysis times and α‐spending function. For the procedures based on RMST, which require $L_{j}\leq t_{j}$, j=1,…,K, at each interim analysis, because of the potential instability of ${\hat{\theta }}_{j}$ at the tail of the distribution, we considered choosing $L_{j}=t_{j},t_{j}-0.1$, and $t_{j}-0.2$. The latter two choices resulted in empirical type I error closest to the nominal 0.05 level with no discernible loss of power against any of the alternatives considered. We thus report results with $L_{j}=t_{j}-0.2$, j=1,…,K.

We consider several group sequential test procedures: that based on the RMST test statistic $n^{1/2}{\hat{\theta }}_{j}/{\left\{{\hat{𝒱}}_{\theta }(j,j)\right\}}^{1/2}$, j=1,…,K; the modified RMST test as in (28) constructed using (26) to favor log odds alternatives, which we refer to as RMST I; the modified RMST test as in (28) constructed using (27) with ${𝒯}_{delay}=0$ to favor proportional hazards alternatives, RMST II; and the modified RMST test as in (28) constructed using (27) with ${𝒯}_{delay}=0.5$ to favor non‐proportional hazards alternatives, RMST III. Because the statistic for the usual RMST test does not have the independent increments property, boundaries are obtained using the above α‐spending function with multivariate normal integration accomplished using the mvtnorm package in R [17], analogous to the construction of boundaries for the adjusted Wilcoxon test in Section 4.2. For the modified tests RMST I–III, we used the ldbounds package in R [18] to compute the boundaries.

Empirical type I error and power under the alternatives are shown in Table 1. All tests based on RMST achieve the nominal level of 0.05, and, as expected, RMST I has the greatest power to detect the log‐odds alternative among them, somewhat less than that for Wilcoxon II, which also targets this alternative. RMST III has substantially greater power for detecting the non‐proportional hazards alternative than all other tests. Interestingly, RMST II has power approaching that of the logrank test for the proportional hazards alternative. Although our focus here is not on the relative performance of these test procedures, the results suggest that basing monitoring of treatment differences on RMST statistics as an omnibus approach may have good properties under a range of alternatives of interest. The Monte Carlo averages of the standardized empirical covariance matrices for the statistics involved in each RMST method under the null hypothesis are shown in Table 2. As expected, that for the unmodified RMST statistic is not consistent with the independent increments property while those for RMST I‐III are. The average number of analyses conducted shows a similar pattern as that for the Wilcoxon tests.

## 5 Practical Implementation

We present the steps an analyst would take to implement the methods in the analysis of a clinical trial in practice. We assume that the analyst has defined the null hypothesis of interest, for example, $H_{0}:S_{1}(u)=S_{0}(u)$, u≥0, or, if expected years of life saved is the treatment effect measure, $H_{0}:\theta =0$, where the RMST difference $\theta =R_{1}(L)-R_{0}(L)=\int_{0}^{L}\{S_{1}(u)-S_{0}(u)\}du$, along with the clinically important alternative to be targeted. The alternative may be chosen based on the particular features of the treatments, as demonstrated in the application in Section 6 or on prior experience, expert knowledge, or convention. These specifications dictate upon which of the methods in Sections 4.2 or 4.3 to base interim analyses. For example, if $H_{0}$ corresponding to RMST is the focus, one would identify the RMST‐based statistic favoring the chosen alternative. The analyst would then specify the number K and times $t_{1},\ldots ,t_{K}$ of potential interim analyses (often based on logistical considerations), the level of significance α, and the α‐spending function characterized by $\left(\alpha_{1},\ldots ,\alpha_{K}\right)$, $\alpha_{1}<\cdots <\alpha_{K}=\alpha$. As is common in practice, the potential sample size n might be determined based on the desired power to detect the chosen alternative at the final (Kth) analysis using standard methods, for example, for RMST [20, 21], taking into account resource and other constraints. Alternatively, the analyst might employ simulations, conducting Monte Carlo trials involving interim analyses using the chosen method under generative scenarios representative of candidate design expectations for the trial, implementing the steps we now present in each simulated trial to determine n based on empirical power.

Given a trial design embodying the above considerations, we outline the steps an analyst would take to implement interim monitoring as the trial progresses, focusing for definiteness on stopping for efficacy as in the simulations in Sections 4.2 and 4.3. The data available at interim analysis time t can be expressed as $\left\{E_{i},Z_{i},U_{i}(t),\Delta_{i}(t)\right\}$ for all subjects i for whom $E_{i}\leq t$, where $U_{i}(t)=U_{i},\Delta_{i}(t)=\Delta_{i}$ if $U_{i}\leq t-E_{i}$, and $U_{i}(t)=t-E_{i}$, $\Delta_{i}(t)=0$ if $U_{i}$ has not yet been observed; that is, $U_{i}(t)=\min(U_{i},t-E_{i}),\Delta_{i}(t)=\Delta_{i}I(U_{i}\leq t-E_{i})$. If the methods based on Gehan's Wilcoxon statistics in Section 4.2 are to be used, define

$$\begin{array}{ll} N(u,t) & =\underset{i:E_{i}\leq t}{\sum }I\{U_{i}(t)\leq u,\Delta_{i}(t)=1\},\\ W(u,t) & =\underset{i:E_{i}\leq t}{\sum }I\{U_{i}(t)\geq u\},\;\bar{Z}(u,t)=\frac{\sum_{i:E_{i}\leq t}Z_{i}I\{U_{i}(t)\geq u)}{W(u,t)}, \end{array}$$

$$\begin{array}{ll} G(t) & =\underset{i:E_{i}\leq t}{\sum }\int_{0}^{t}W(u,t)\{Z_{i}-\bar{Z}(u,t)\}dN(u,t),\\ \hat{𝒱}(s,t) & =\int_{0}^{s}W(u,s)W(u,t)\bar{Z}(u,s)\{Z_{i}-\bar{Z}(u,s)\}dN(u,s); \end{array}$$

and, with $\hat{S}(u,t)$ the Kaplan–Meier estimator of S(u) under $H_{0}$ based on the available data from both treatments through time t,

(29) $$\begin{array}{ll} {\hat{\mu }}_{lo}(t) & =-\int_{0}^{t}W(u,t)\bar{Z}(u,t)\{Z_{i}-\bar{Z}(u,t)\}\hat{S}(u,t)dN(u,t),\\ {\hat{\mu }}_{ph}(t) & =-\int_{0}^{t}W(u,t)\bar{Z}(u,t)\{Z_{i}-\bar{Z}(u,t)\}dN(u,t),\\ {\hat{\mu }}_{nph}(t) & =-\int_{{𝒯}_{delay}}^{t}W(u,t)\bar{Z}(u,t)\{Z_{i}-\bar{Z}(u,t)\}dN(u,t), \end{array}$$

corresponding to the log odds, proportional hazards, and non‐proportional hazards alternatives, where ${𝒯}_{delay}$ is as defined previously. Likewise, if the methods based on RMST in Section 4.3 are to be used, define the treatment‐specific quantities

$$\begin{array}{ll} N_{z}(u,t) & =\underset{i:E_{i}\leq t}{\sum }I\{U_{i}(t)\leq u,\Delta_{i}(t)=1,Z_{i}=z\},\\ W_{z}(u,t) & =\underset{i:E_{i}\leq t}{\sum }I\{U_{i}(t)\geq u,Z_{i}=z\},z=0,1, \end{array}$$

and define

$$\begin{array}{ll} \hat{\theta }\{t,L(t)\} & =\int_{0}^{L(t)}\{{\hat{S}}_{1}(u,t)-{\hat{S}}_{0}(u,t)\}\;du,{\hat{𝒱}}_{\theta }(s,t)\\ & =\overset{1}{\underset{z=0}{\sum }}\left[\int_{0}^{L(s)}\frac{{\hat{A}}_{z}\{u,s,L(s)\}{\hat{A}}_{z}\{u,t,L(t)\}\;dN_{z}(u,t)}{W_{z}(u,t)\{W_{z}(u,t)-dN_{z}(u,t)\}}\right], \end{array}$$

where L(t) is the restricted time corresponding to t, ${\hat{S}}_{z}(u,t)$ the Kaplan–Meier estimator of $S_{z}(u)$ based on the available data through time t, and

$${\hat{A}}_{z}\{u,t,L(t)\}=\int_{u}^{L(t)}{\hat{S}}_{z}(x,t)\;dx.$$

Further, with $\hat{\Lambda }(u,t)$ the Nelson‐Aalen estimator for the cumulative hazard function under $H_{0}$ using the available data from both treatments through time t and L(t) the corresponding restricted time, define analogous to (29)

(30) $$\begin{array}{ll} {\hat{\mu }}_{lo}\{t,L(t)\} & =\int_{0}^{L(t)}\hat{S}(u,t)\{1-\hat{S}(u,t)\}\;du,\\ {\hat{\mu }}_{ph}\{t,L(t)\} & =\int_{0}^{L(t)}\hat{S}(u,t)\hat{\Lambda }(u,t)\;du,\\ {\hat{\mu }}_{nph}\{t,L(t)\} & =\int_{{𝒯}_{delay}}^{L(t)}\hat{S}(u,t)\{\hat{\Lambda }(u,t)-\hat{\Lambda }({𝒯}_{delay},t)\}\;du. \end{array}$$

Given these definitions, the steps are as follows.

1. At the first interim analysis time $t_{1}$, based on the available data at $t_{1}$, if methods based on Gehan's Wilcoxon statistic are being used, calculate $X_{1}=G(t_{1})$, ${\hat{\sum }}_{1}=\hat{𝒱}(t_{1},t_{1})$, and ${\hat{\mu }}_{1}=\hat{\mu }(t_{1})$, where $\hat{\mu }(t)$ is the appropriate choice in (29) for the targeted alternative of interest. If methods based on RMST are being used, calculate $X_{1}=\hat{\theta }\{t_{1},L(t_{1})\}$; ${\hat{\sum }}_{1}={\hat{𝒱}}_{\theta }(t_{1},t_{1})$; and ${\hat{\mu }}_{1}=\hat{\mu }\{t_{1},L_{1}(t_{1})\}$, where $\hat{\mu }\{t,L(t)\}$ is the appropriate choice in (30) for the targeted alternative, and $L_{1}=L(t_{1})$. Then form the standardized test statistic ${𝕋}_{1}=X_{1}/\;{\hat{\sum }}_{1}^{1/2}$ and reject $H_{0}$ if ${𝕋}_{1}$ or $\left|{𝕋}_{1}\right|$ exceeds the boundary value $\zeta_{1}=z_{\alpha_{1}}$ or $z_{\alpha_{1}/2}$, respectively, where $z_{\eta }$is the (1−η)th quantile of the standard normal distribution. If $H_{0}$ is rejected, stop; otherwise calculate ${\hat{v}}_{1}={\hat{\mu }}_{1}^{2}{\hat{\sum }}_{1}^{-1}$ and continue to step 2.
2. At the second interim analysis time $t_{2}$, based on the available data at $t_{2}$, if methods based on Gehan's Wilcoxon statistics are being used, calculate $X_{2}=G(t_{2})$,
(31) $${\hat{\sum }}_{2}=\left(\begin{array}{cc} \hat{𝒱}(t_{1},t_{1}) & \hat{𝒱}(t_{1},t_{2})\\ \hat{𝒱}(t_{1},t_{2}) & \hat{𝒱}(t_{2},t_{2}) \end{array}\right),$$
  and ${\underline{\hat{\mu }}}_{2}={\left\{\hat{\mu }(t_{1}),\hat{\mu }(t_{2})\right\}}^{T}$, where $\hat{\mu }(t)$ is the appropriate choice in (29) for the targeted alternative of interest. If methods based on RMST are being used, calculate $X_{2}=\hat{\theta }\{t_{2},L(t_{2})\}$, with
(32) $${\hat{\sum }}_{2}=\left(\begin{array}{cc} {\hat{𝒱}}_{\theta }(t_{1},t_{1}) & {\hat{𝒱}}_{\theta }(t_{1},t_{2})\\ {\hat{𝒱}}_{\theta }(t_{1},t_{2}) & {\hat{𝒱}}_{\theta }(t_{2},t_{2}) \end{array}\right);$$
  and 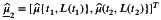, where $\hat{\mu }\{t,L(t)\}$ is the appropriate choice in (30) for the targeted alternative, and $L_{2}=L(t_{2})$. Form ${\underline{X}}_{2}={\left(X_{1},X_{2}\right)}^{T}$, calculate $Y_{2}={\underline{\hat{\mu }}}_{2}^{T}{\hat{\sum }}_{2}^{-1}{\underline{X}}_{2}$, ${\hat{v}}_{2}={\underline{\hat{\mu }}}_{2}^{T}{\hat{\sum }}_{2}^{-1}{\underline{\hat{\mu }}}_{2}$, and form the standardized test statistic ${𝕋}_{2}=Y_{2}/\;{\hat{v}}_{2}^{1/2}$. Calculate the proportion of information achieved at $t_{1}$ and $t_{2}$ as ${ℐ}_{1}={\hat{v}}_{1}/\;{\hat{v}}_{2}$ and ${ℐ}_{2}={\hat{v}}_{2}/\;{\hat{v}}_{2}$, respectively, and, using standard software, for example, the R package ldbounds, based on $\left({ℐ}_{1},{ℐ}_{2}\right)$, obtain the boundary $\zeta_{2}$ for a one‐ or two‐sided test as appropriate, where the α‐spending function is characterized by $\left(\alpha_{1},\alpha_{2}\right)$. Reject $H_{0}$ if ${𝕋}_{2}$ or $\left|{𝕋}_{2}\right|$ exceeds the boundary value $\zeta_{2}$. If $H_{0}$ is rejected, stop; otherwise continue to Step 3.
3. For j=3,…,K, if the procedure failed to reject $H_{0}$at $t_{j-1}$, based on the available data at $t_{j}$, if methods based on Gehan's Wilcoxon statistics are being used, calculate $X_{j}=G(t_{j})$,
(33) $${\hat{\sum }}_{j}=\left(\begin{array}{ccc} \hat{𝒱}(t_{1},t_{1}) & \cdots & \hat{𝒱}(t_{1},t_{j})\\ \hat{𝒱}(t_{1},t_{2}) & \cdots & \hat{𝒱}(t_{2},t_{j})\\ \vdots & \ddots & \vdots \\ \hat{𝒱}(t_{1},t_{j}) & \cdots & \hat{𝒱}(t_{j},t_{j}) \end{array}\right),$$
  and ${\underline{\hat{\mu }}}_{j}={\left\{\hat{\mu }(t_{1}),\ldots ,\hat{\mu }(t_{j})\right\}}^{T}$, where $\hat{\mu }(t)$ is the appropriate choice in (29) for the targeted alternative of interest. If methods based on RMST are being used, and calculate $X_{j}=\hat{\theta }\{t_{j},L(t_{j})\}$,
(34) $${\hat{\sum }}_{j}=\left(\begin{array}{ccc} {\hat{𝒱}}_{\theta }(t_{1},t_{1}) & \cdots & {\hat{𝒱}}_{\theta }(t_{1},t_{j})\\ {\hat{𝒱}}_{\theta }(t_{1},t_{2}) & \cdots & {\hat{𝒱}}_{\theta }(t_{2},t_{j})\\ \vdots & \ddots & \vdots \\ {\hat{𝒱}}_{\theta }(t_{1},t_{j}) & \cdots & {\hat{𝒱}}_{\theta }(t_{j},t_{j}) \end{array}\right),$$
  and ${\underline{\hat{\mu }}}_{j}={\left[\hat{\mu }\{t_{1},L(t_{1})\},\ldots ,\hat{\mu }\{t_{j},L(t_{j})\}\right]}^{T}$, where $\hat{\mu }\{t,L(t)\}$ is the appropriate choice in (30) for the targeted alternative, and $L_{j}=L(t_{j})$. Form ${\underline{X}}_{j}={\left(X_{1},\ldots ,X_{j}\right)}^{T}$, calculate $Y_{j}={\underline{\hat{\mu }}}_{j}^{T}{\hat{\sum }}_{j}^{-1}{\underline{X}}_{j}$, ${\hat{v}}_{j}={\underline{\hat{\mu }}}_{j}^{T}{\hat{\sum }}_{j}^{-1}{\underline{\hat{\mu }}}_{j}$, and form the standardized test statistic ${𝕋}_{j}=Y_{j}/\;{\hat{v}}_{j}^{1/2}$. Calculate the proportion of information achieved at $t_{1},t_{2},\ldots ,t_{j}$ as ${ℐ}_{1}={\hat{v}}_{1}/\;{\hat{v}}_{j}$, ${ℐ}_{2}={\hat{v}}_{2}/\;{\hat{v}}_{j}$, $\ldots ,{ℐ}_{j}={\hat{v}}_{j}/\;{\hat{v}}_{j}$, respectively, and, using standard software, for example, the R package ldbounds, based on $\left({ℐ}_{1},\ldots ,{ℐ}_{j}\right)$, obtain the boundary $\zeta_{j}$ for a one‐ or two‐sided test as appropriate, where the α‐spending function is characterized by $\left(\alpha_{1},\alpha_{2},\ldots ,\alpha_{j}\right)$. Reject $H_{0}$ if ${𝕋}_{j}$ or $\left|{𝕋}_{j}\right|$ exceeds the boundary value $\zeta_{j}$. If $H_{0}$ is rejected, stop; otherwise, if j<K, let j=j+1 and repeat Step 3; if j=K and $H_{0}$ is not rejected, conclude that there is not sufficient evidence from the trial to reject $H_{0}$.

In the Supporting Information, we demonstrate how we specified the α‐spending function and obtained stopping boundaries using the R package ldbounds in the simulations in Sections 4.2 and 4.3 and in the application discussed in the next section.

## 6 Application: North American Leukemia Intergroup Study C9710

We demonstrate use of the methods in practice by applying them retroactively to data from North American Leukemia Intergroup Study C9710, coordinated by the Cancer and Leukemia Group B, now part of the Alliance for Clinical Trials in Oncology, in patients with acute promyelocytic leukemia (APL) [22], for which the primary outcome was (possibly censored) event‐free survival (EFS) time, a composite of time to failure to achieve complete remission (CR), relapse after CR, or death, whichever comes first. APL patients were randomized to receive standard induction chemotherapy, all‐trans‐retinoic acid (ATRA), followed by ATRA consolidation therapy with or without addition of arsenic trioxide (As_2_O_3_) if CR was achieved during/after induction. Patients remaining in CR after completion of consolidation were re‐randomized to one of two maintenance regimens; we focus here on the first randomization only. Thus, all patients received up to 90 days of the same ATRA induction therapy followed by assigned consolidation (ATRA or ATRA+As_2_O_3_) if CR was achieved. Consequently, it is natural to expect a delayed treatment effect (comparison of EFS distributions for ATRA with ATRA versus ATRA+As_2_O_3_ consolidation) of up to 90 days.

The analysis reported by Powell et al. [22] was completed on December 1, 2008 (day 3360 from the start of enrollment). Because the data provided to us have more extensive follow up, we created an illustrative analysis data set by censoring EFS outcomes at this date and eliminating seven patients with missing or anomalous values (e.g., enrollment date missing or after induction therapy start date), leaving data on 474 patients, of whom 234 (240) were randomized to ATRA (ATRA+As_2_O_3_) consolidation following ATRA induction, who enrolled in the trial starting in 1999. About 90% of patients in each group achieved CR. Powell et al. state that interim analyses of EFS were conducted semiannually using the logrank test; accordingly, we consider possible interim analyses every 3360/18 ≈187 days through day 3360. Owing to the paucity of events early in the trial, for illustration, we commence formal interim analyses at the third time (day 560 from start of enrollment), with 15 additional planned analyses. Given the expected delayed treatment effect, we chose a priori to base analyses on the modified RMST test statistic as in (28) constructed using (27) with ${𝒯}_{delay}=90$ favoring non‐proportional hazards alternatives, denoted as RMST III in Section 4.3, consistent with current interest in RMST as an alternative to the logrank test under such conditions. At each interim time $t_{j}$, we take $L_{j}=t_{j}-5$ days. For both RMST III and the logrank test, which have the independent increments property, for two‐sided tests with level of significance α=0.05, we used the R package ldbounds [18] to obtain the O'Brien‐Fleming α‐spending function corresponding to the time since start of enrollment for each of the K=16 planned analysis times; that is, $(t_{1},\ldots ,t_{16})/t_{16}$ in the ldBounds() function; see the Supporting Information. We then obtained the stopping boundaries as in Steps 1–3 of the Section 5.

**FIGURE 1:**
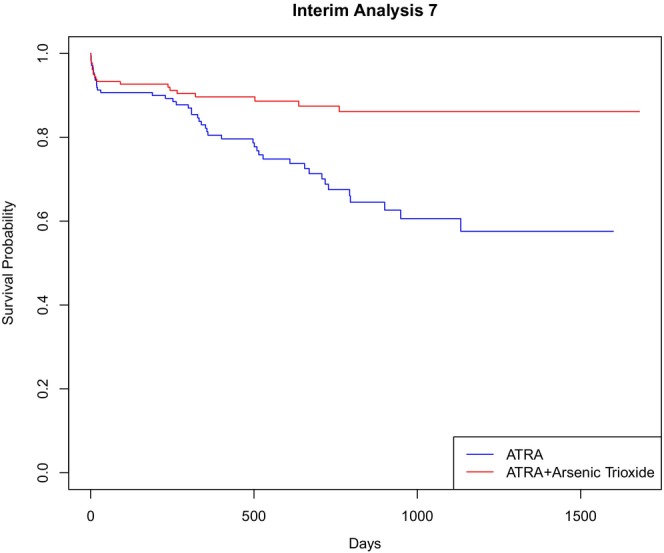
Treatment‐specific Kaplan–Meier estimates of EFS distributions at interim analysis 7.

Table 3 presents the values of the standardized test statistics and the associated stopping boundaries to which their absolute values are compared at the first 10 potential interim analyses. Both standardized test statistics in absolute value exceed the stopping boundary for the first time at the seventh interim analysis at day 1680, at which point 66 events had occurred, 20 and 46 in the ATRA and ATRA+As_2_O_3_ groups, respectively. Notably, the modified RMST test statistic is consistently larger than the logrank statistic at all analyses and achieves an impressively large value relative to its corresponding boundary at the seventh analysis (and subsequently) and comes close to crossing the boundary at the sixth analysis, presumably reflecting that it is designed to target non‐proportional hazards alternatives. Figure 1 shows the Kaplan–Meier estimates of EFS distributions at interim analysis 7, which are consistent with both a treatment effect delay and evolving strong evidence of a treatment difference, aligned with the ultimate finding of Powell et al.

**TABLE 3:** Standardized modified RMST test statistics with ${𝒯}_{delay}=90$ favoring non‐proportional alternatives (RMST III) and logrank test statistics and associated stopping boundaries for retrospective interim analyses of the Study C9710 data.

	Interim analysis time (days)									
	560	746	933	1120	1310	1490	1680	1870	2050	2240
RMST III										
Statistic^a^	1.701	2.034	2.882	2.449	2.288	3.041	**4.340**	4.870	4.636	4.863
Boundary	5.367	4.607	4.109	3.750	3.445	3.258	**3.100**	2.923	2.767	2.621
Logrank										
Statistic	−1.639	−1.820	−2.660	−2.483	−2.103	−2.609	**‐3.568**	−3.913	−3.736	−4.050
Boundary	5.367	4.622	4.104	3.742	3.458	3.246	**3.057**	2.896	2.767	2.616

*Note:* Boldface indicates stopping boundary crossed.

^a^ Value of standardized test statistic.

## 7 Discussion

There is a vast literature on monitoring treatment differences in randomized clinical trials using group sequential tests. Much of the methodology is based on the premise that the associated statistics have the independent increments property, which allows standard algorithms and software to be used to compute stopping boundaries. However, this property may not hold for some tests of interest in practice. We have demonstrated that, regardless of whether or not the covariance matrix of the relevant statistics has the independent increments structure, it is always possible to find linear combinations of them that do have this structure. Moreover, we show how the linear combinations can be chosen judiciously to result in tests with high power to detect specific alternatives of interest. Thus, modified test statistics that can be derived from our results will have improved power, and monitoring based on them can be implemented readily using existing group sequential software.

## Conflicts of Interest

The authors declare no conflicts of interest.

## Supporting information




**Data S1.** Supporting Information.

## Data Availability

The data used for illustration in this article were obtained under a data sharing agreement with the Alliance for Clinical Trials in Oncology and cannot be shared by the authors.
