# Supplementary material for: Independent Increments and Group Sequential Tests
Source: Stat Med. 2025 Nov 7;44(25-27):e70307. doi: 10.1002/sim.70307 (PMC12593325; doi:10.1002/sim.70307)
Supplement: Supplementary file 1 — Data S1. Supporting Information. [file SIM-44-0-s001.zip › supporting.pdf]

Received: Added at production | Revised: Added at production | Accepted: Added at production
DOI: xxx/xxxx

RESEARCH ARTICLE

Supporting Information for "Independent increments and group sequential tests"

Anastasios A. Tsiatis | **Marie Davidian**

[1]Department of Statistics, North Carolina State University, North Carolina, USA

Correspondence
Corresponding author Marie Davidian,
Email: davidian@ncsu.edu

Present address
Department of Statistics, North Carolina State University, Raleigh, North Carolina, USA..

A | PROOF OF THEOREM 1

Throughout, notation is as defined in the main paper. For convenience, we restate Theorem 1, followed by the proof.

Theorem 1. *Under the above conditions, with $\mathcal{V}$ of full rank and $\mathcal{V}_j$, $j = 1, \ldots, K$, defined as above, the vector of nontrivial, sequentially-computed linear combinations $Y = (Y_1, \ldots, Y_K)^T$ has the independent increments property if and only if there exists a vector of constants $b = (b_1, \ldots, b_K)^T$ such that $a_j = \mathcal{V}_j^- \underline{b}_j$, where $\underline{b}_j = (b_1, \ldots, b_j, 0, \ldots, 0)^T$, $j = 1, \ldots, K$, and $\mathcal{V}_j^-$ is the $(K \times K)$ matrix with upper left hand $(j \times j)$ submatrix the inverse of the covariance matrix of $(X_1, \ldots, X_j)^T$ and all remaining elements of the matrix equal to zero; i.e., $\mathcal{V}_j^-$ is the Moore-Penrose generalized inverse of $\mathcal{V}_j$.*

Proof. First, assume that $a_j = \mathcal{V}_j^- \underline{b}_j$ holds. It is straightforward that, in general, $\mathcal{V}_Y(j,k) = a_j^T \mathcal{V} a_k$, $j,k = 1, \ldots, K$; thus, it follows that $\mathcal{V}_Y(j,k) = \underline{b}_j^T \mathcal{V}_j^- \mathcal{V} \mathcal{V}_k^- \underline{b}_k$. However, for $j \leq k$, $\mathcal{V}_j^- \mathcal{V} \mathcal{V}_k^- = \mathcal{V}_j^-$, so that $\mathcal{V}_Y(j,k) = \underline{b}_j^T \mathcal{V}_j^- \underline{b}_k = \underline{b}_j^T \mathcal{V}_j^- \underline{b}_j$. Thus, $\mathcal{V}_Y(j,k) = \mathcal{V}_Y(j,j)$, $j \leq k, j,k = 1, \ldots, K$, so that Y has the independent increments property.

Conversely, assume that $Y = (Y_1, \ldots, Y_K)^T$ has the independent increments property. We wish to show that there exists a vector $b = (b_1, \ldots, b_K)^T$ such that $a_j = \mathcal{V}_j^- \underline{b}_j$ for $j = 1, \ldots, K$. Define $q_j = \mathcal{V}_j a_j$, $j = 1, \ldots, K$, in which case $a_j = \mathcal{V}_j^- q_j$. Note that, because $\mathcal{V}$ is of full rank, the $(j \times j)$ upper left submatrix of $\mathcal{V}$ has a unique inverse, which is the $(j \times j)$ upper left submatrix of $\mathcal{V}_j^-$. We now show that if Y, derived using nontrivial linear combinations, has the independent increments property, then if we take $b_j = q_{jj}$, the jth element of $q_j = \mathcal{V}_j a_j$, then a_j must equal $\mathcal{V}_j^- \underline{b}_j$, which proves the converse. As above, for $\ell \leq \ell'$,

$$\mathcal{V}_Y(\ell, \ell') = a_\ell^T \mathcal{V} a_{\ell'} = q_\ell^T \mathcal{V}_\ell^- \mathcal{V} \mathcal{V}_{\ell'}^- q_{\ell'} = q_\ell^T \mathcal{V}_\ell^- q_{\ell'} = a_\ell^T q_{\ell'}. \tag{A1}$$

The proof is by induction. Suppose that it has already been shown that that $q_\ell = \underline{b}_\ell$, where $\underline{b}_\ell = (b_1, \ldots, b_\ell, 0, \ldots, 0)^T$, and $b_\ell = q_{\ell\ell}$, $\ell = 1, \ldots, j-1$. Then we must show that $q_j = \underline{b}_j$, or $q_{j1} = b_1, \ldots, q_{j,j-1} = b_{j-1}$. Because Y has the independent increments property, $\mathcal{V}_Y(\ell, \ell') = \mathcal{V}_Y(\ell, \ell)$, $\ell \leq \ell', \ell, \ell' = 1, \ldots, K$, which, by (A1), implies that

$$a_\ell^T q_{\ell'} = a_\ell^T q_\ell. \tag{A2}$$

Take $\ell = 1$ and $\ell' = j$. Then (A2) implies that $a_{11} q_{j1} = a_{11} q_{11}$, and because $a_{11} \neq 0$, it follows that $q_{j1} = q_{11} = b_1$. Next, take $\ell = 2$ and $\ell' = j$. Then (A2) implies that $a_{21} q_{j1} + a_{22} q_{j2} = a_{21} q_{21} + a_{22} q_{22}$. We already showed that $q_{j1} = q_{11}$, and, by assumption.

 © 2023 Copyright Holder Name

$q_{21} = b_1$. Thus, $a_{21}b_1 + a_{22}q_{j2} = a_{21}b_1 + a_{22}q_{22}$. Because $a_{22} \neq 0$, it follows that $q_{j2} = q_{22} = b_2$. Continuing in this fashion, we obtain $q_{j\ell} = b_\ell$, $\ell = 1, \ldots, j-1$, and finally $q_{jj} = b_j$, completing the proof. □

B | OPTIMALITY OF THE TEST IN SECTION 3.1

We show that the test procedure based on the standardized test statistics

$$Y^{opt}_{j,n} / (\underline{\mu}_j^T \mathcal{V}_j^- \underline{\mu}_j)^{1/2}, \quad j = 1, \ldots, K,$$

in (4) of the main paper is the most powerful that can be constructed based on $X_{1,n}, \ldots, X_{K,n}$ for a specified level of significance α and α-spending function, including relative to tests that are not based on linear combinations of $X_{1,n}, \ldots, X_{K,n}$.

For definiteness, from (3) of the main paper, take

$$Y^{opt}_{j,n} = \underline{\mu}_j^T \mathcal{V}_j^- \underline{X}_{j,n}.$$

As in the main paper, for $j = 1, \ldots, K$, under H_0,

$$Y^{opt}_{j,n} \dot{\sim} N(0, \Sigma_j), \quad \Sigma_j = \underline{\mu}_j^T \mathcal{V}_j^- \underline{\mu}_j.$$

Similarly, under $H_{A,n}$,

$$Y^{opt}_{j,n} \dot{\sim} N(\Sigma_j, \Sigma_j).$$

By construction, $Y^{opt}_n = (Y^{opt}_{1,n}, \ldots, Y^{opt}_{K,n})^T$ has the independent increments property.

Define

$$\begin{aligned}
\Delta Y^{opt}_{1,n} &= Y^{opt}_{1,n} \\
\Delta Y^{opt}_{2,n} &= Y^{opt}_{2,n} - Y^{opt}_{1,n} \\
&\vdots \\
\Delta Y^{opt}_{K,n} &= Y^{opt}_{K,n} - Y^{opt}_{K-1,n}.
\end{aligned}$$

By the independent increments property and the asymptotic normality above, $\Delta Y^{opt}_{1,n}, \ldots, \Delta Y^{opt}_{K,n}$ are asymptotically independent, and, under H_0,

$$\Delta Y^{opt}_{1,n} \dot{\sim} N(0, \Sigma_1),$$

$$\Delta Y^{opt}_{j,n} \dot{\sim} N(0, \Sigma_j - \Sigma_{j-1}), \quad j = 2, \ldots, K.$$

Let $\Delta\Sigma_j = \Sigma_j - \Sigma_{j-1}$. According to Theorem 1 of Tsiatis and Mehta[1], among all sequential tests based on $(\Delta Y^{opt}_{1,n}, \ldots, \Delta Y^{opt}_{K,n})$ that use a prespecified α-spending function, the optimal such test is based on the sequential likelihood ratio test statistic

$$\prod_{\ell=1}^{j} LR_\ell, \tag{B1}$$

where the likelihood ratio for $\Delta Y^{opt}_{\ell,n}$, LR_ℓ, $\ell = 1, \ldots, j$, is given by

$$\begin{aligned}
LR_\ell &= \frac{(2\pi\Delta\Sigma_\ell)^{-1/2} \exp\{-(\Delta Y^{opt}_{\ell,n} - \Delta\Sigma_\ell)^2/(2\Delta\Sigma_\ell)\}}{(2\pi\Delta\Sigma_\ell)^{-1/2} \exp\{-(\Delta Y^{opt}_{\ell,n})^2/(2\Delta\Sigma_\ell)\}} \\
&= \exp\{\Delta Y^{opt}_{\ell,n} - \Delta\Sigma_\ell/2\}.
\end{aligned}$$

Thus,

$$\begin{aligned}\prod_{\ell=1}^{j} LR_\ell &= \prod_{\ell=1}^{j} \exp\{\Delta Y_{\ell,n}^{opt} - \Delta\Sigma_\ell/2\} \\ &= \exp\left\{\sum_{\ell=1}^{j} \Delta Y_{\ell,n}^{opt} - \sum_{\ell=1}^{j} \Delta\Sigma_\ell/2\right\} \\ &= \exp\{Y_{j,n}^{opt} - \Sigma_j/2\}.\end{aligned}$$

The sequential likelihood ratio test rejects H_0 at the jth interim analysis when the statistic (B1) exceeds the critical value determined by the α-spending function, ζ_j, say. That is, when

$$\exp\{Y_{j,n}^{opt} - \Sigma_j/2\} > \zeta_j,$$

or equivalently when $Y_{j,n}^{opt}$ exceeds a constant. Consequently, the optimal test has higher power than any other sequential test that is based on $\Delta Y_{1,n}^{opt}, \ldots, \Delta Y_{j,n}^{opt}$ and uses that same level of significance α and α-spending function, including any test based on linear combinations of $X_{1,n}, \ldots, X_{j,n}$, because such linear combinations are also linear combinations of $\Delta Y_{1,n}^{opt}, \ldots, \Delta Y_{j,n}^{opt}, j = 1, \ldots, K$.

Strictly speaking, the foregoing developments apply to one-sided tests. Given that a two-sided test can be viewed as roughly comprising two one-sided tests, each at level $\alpha/2$, the implications of this result should carry over approximately to two-sided tests.

C | TECHNICAL DETAILS: GEHAN'S WILCOXON STATISTICS

Variance and covariance of influence functions. To obtain var$\{IF(t)\}$ given in (7) of the main paper, by standard counting process martingale results, and absorbing $I(E \leq t)$ into $I(U \geq u, t - E \geq u)$ for all $u \geq 0$,

$$\begin{aligned}\text{var}\{IF(t)\} &= E\left\{\int_0^t w^2(u,t)\{Z - \pi(u,t)\}^2 I(U \geq u, t-E \geq u)\lambda(u)du\right\} \\ &= \int_0^t w^2(u,t)\pi(u,t)\{1-\pi(u,t)\}E\{I(U \geq u, t-E \geq u)\}\lambda(u)du = \int_0^t w^3(u,t)\pi(u,t)\{1-\pi(u,t)\}\lambda(u)du, \quad \text{(C1)}\end{aligned}$$

which is (7) of the main paper, where, under H_0, (C1) follows by taking the expectation inside the integral. Similarly, for $s \leq t$,

$$\begin{aligned}\text{cov}\{IF(s), IF(t)\} &= E\left\{\int_0^s w(u,s)w(u,t)\{Z-\pi(u,t)\}\{Z-\pi(u,s)\}I(T \geq u, s-E \geq u)\lambda(u)du\right\} \\ &= \int_0^s w(u,s)w(u,t)\pi(u,s)\{1-\pi(u,s)\}E\{I(T \geq u, s-E \geq u)\}\lambda(u)du = \int_0^s w^2(u,s)w(u,t)\pi(u,s)\{1-\pi(u,s)\}\lambda(u)du,\end{aligned}$$

which is (8) of the main paper.

Asymptotic mean of Gehan's Wilcoxon statistics under log-odds alternative. We give a heuristic argument to show that, under local log-odds alternatives, the asymptotic mean of the statistic used to construct Gehan's Wilcoxon is given by (12) of the main paper. Under the log-odds alternative (11) of the main paper, the corresponding hazard functions can be found by taking minus the derivative of the log survival functions, which yields

$$\lambda_1(u,\delta) = \lambda_0(u) + \frac{\{\exp(\delta)-1\}\lambda_0(u)S_0(u)}{1 + S_0(u)\{\exp(\delta)-1\}}.$$

Under local alternatives δ_n such that $n^{1/2}\delta_n \to \tau$,

$$\lambda_1(u,\delta_n) = \lambda_0(u)\{1 - \delta_n S_0(u)\} + o(\delta_n), \quad \text{(C2)}$$

where $o(\delta_n)$ is a term that is of small order in δ_n; i.e., $o(\delta_n)/\delta_n \to 0$. By (6) of the main paper, the sequentially-computed statistic $G_n(t)$ is asymptotically equivalent to

$$n^{-1/2}\sum_{i=1}^{n}\int_0^t w(u,t)\{Z_i - \pi(u,t)\}d\mathcal{M}_i(u)I(t-E_i \geq u) \tag{C3}$$

under the null hypothesis. Because of contiguity, the asymptotic equivalence is also true under the local alternatives. However, for local alternatives, the martingale increment process is given by

$$d\mathcal{M}_i(u,\delta_n) = d\mathcal{N}_i(u) - \{\lambda_1(u,\delta_n)Z_i + \lambda_0(u)(1-Z_i)\}I(T_i \geq u)du,$$

which has mean zero under the sequence of local alternatives. We write (C3) as

$$n^{-1/2}\sum_{i=1}^{n}\int_0^t w(u,t)\{Z_i - \pi(u,t)\}d\mathcal{M}_i(u,\delta_n)I(t-E_i \geq u) \tag{C4}$$

$$+\ n^{-1/2}\sum_{i=1}^{n}\int_0^t w(u,t)\{Z_i - \pi(u,t)\}\{d\mathcal{M}_i(u) - d\mathcal{M}_i(u,\delta_n\}I(t-E_i \geq u). \tag{C5}$$

Under local alternatives, (C4) converges to the same distribution as (C3) does under the null hypothesis; namely $N\,[0, \text{var}\{IF(t)\}]$. Now

$$d\mathcal{M}_i(u) - d\mathcal{M}_i(u,\delta_n) = Z_i\{\lambda_1(u,\delta_n) - \lambda_0(u)\}I(T_i \geq u),$$

which, using (C2), equals $-Z_i\delta_n\lambda_0(u)S_0(u)I(T_i \geq u) + o(\delta_n)$. Therefore, (C5) is equal to

$$-n^{-1/2}\sum_{i=1}^{n}\int_0^t w(u,t)\{Z_i - \pi(u,t)\}\{Z_i\delta_n\lambda_0(u)S_0(u)I(T_i \geq u) + o(\delta_n)\}I(t-E_i \geq u)du$$

$$= -n^{-1}\sum_{i=1}^{n}\int_0^t w(u,t)\{Z_i - \pi(u,t)\}\{Z_i n^{1/2}\delta_n\lambda_0(u)S_0(u)I(T_i \geq u) + n^{1/2}o(\delta_n)\}I(t-E_i \geq u)du.$$

Because $n^{1/2}\delta_n \to \tau$ and $n^{1/2}o(\delta_n) \to 0$, (C5) is equal to

$$-n^{-1}\sum_{i=1}^{n}\int_0^t w(u,t)\{Z_i - \pi(u,t)\}Z_i\tau\lambda_0(u)S_0(u)I(T_i \geq u)I(t-E_i \geq u)du + o_P(1). \tag{C6}$$

Again, by contiguity, (C6) converges under the local alternatives to the same limit as it would under the null hypothesis, which, by a simple application of the law of large numbers, is equal to

$$-E_{H_0}\left\{\int_0^t w(u,t)\{Z_i - \pi(u,t)\}Z_i\tau\lambda_0(u)S_0(u)I(T_i \geq u, t-E_i \geq u)du\right\} = -\tau\int_0^t w^2(u,t)\pi(u,t)\{1-\pi(u,t)\}\lambda_0(u)S_0(u)du,$$

which is the same as $\mu(t)$ in (12) of the main paper.

Asymptotic mean of Gehan's Wilcoxon statistic under delayed proportional hazards alternative. Following a similar argument as that for the log odds alternative above, under the delayed proportional hazards alternative (15) of the main paper and under local alternatives δ_n such that $n^{1/2}\delta_n \to \tau$,

$$d\mathcal{M}_i(u) - d\mathcal{M}_i(u,\delta_n) = -Z_i\delta_n\lambda_0(u)I(T_i \geq u) + o(\delta_n).$$

Thus, (C5) is equal to

$$
\begin{aligned}
&-n^{-1/2}\sum_{i=1}^{n}\int_0^t w(u,t)\{Z_i-\pi(u,t)\}\{Z_i\delta_n\lambda_0(u)I(u\geq\mathcal{T}_{delay})I(T_i\geq u)+o(\delta_n)\}I(t-E_i\geq u)du\\
&\qquad=-n^{-1}\sum_{i=1}^{n}\int_{\mathcal{T}_{delay}}^t w(u,t)\{Z_i-\pi(u,t)\}\{Z_i n^{1/2}\delta_n\lambda_0(u)I(T_i\geq u)+n^{1/2}o(\delta_n)\}I(t-E_i\geq u)du\\
&\qquad=-n^{-1}\sum_{i=1}^{n}\int_{\mathcal{T}_{delay}}^t w(u,t)\{Z_i-\pi(u,t)\}Z_i\tau\lambda_0(u)I(T_i\geq u)I(t-E_i\geq u)du+o_P(1) \qquad \text{(C7)}
\end{aligned}
$$

because $n^{1/2}\delta_n\to\tau$ and $n^{1/2}o(\delta_n)\to 0$. Again, by contiguity, (C7) converges under the local alternatives to the same limit as it would under the null hypothesis, which by the law of large numbers is equal to

$$
-E\left\{\int_{\mathcal{T}_{delay}}^t w(u,t)\{Z_i-\pi(u,t)\}Z_i\tau\lambda_0(u)I(T_i\geq u,t-E_i\geq u)du\right\}=-\tau\int_{\mathcal{T}_{delay}}^t w^2(u,t)\pi(u,t)\{1-\pi(u,t)\}\lambda_0(u)du,
$$

the same as (16) of the main paper.

D | TECHNICAL DETAILS: RMST-BASED STATISTICS

Derivation of influence functions for $\widehat{R}_z(t,L)$, $z=0,1$. To derive $IF_{R,z,i}(t,L)$, the ith influcence function for $\widehat{R}_z(t,L)$, $z=0,1$, note that the ith influence function for the treatment-specific Kaplan-Meier estimator $\widehat{S}_z(u,t)$ of $S_z(u)$, $IF_{z,i}(u,t)$, say, is defined such that

$$
n_z^{1/2}\{\widehat{S}_z(u,t)-S_z(u)\}=n_z^{-1/2}\sum_{i=1}^{n_z}IF_{z,i}(u,t)+o_P(1).
$$

Standard results for the Kaplan-Meier estimator using counting process methodology yield

$$
IF_{z,i}(u,t)=I(E_{z,i}\leq t)S_1(u)\int_0^u\frac{d\mathcal{M}_{z,i}(x)I(t-E_{z,i}\geq x)}{w_z(x,t)},
$$

where $\mathcal{N}_z(u)=I(U_z\leq u,\Delta_z=1)$, $\mathcal{Y}_z(u)=I(U_z\geq u)$, $d\mathcal{M}_z(u)=d\mathcal{N}_z(u)-\lambda(u)\mathcal{Y}_z(u)du$, and $w_z(u,t)=E\{I(U_{z,i}\geq u,t-E_{z,i}\geq u)\}$, and thus the ith influence function of $\widehat{R}_z(t,L)$ is given by

$$
IF_{R,z,i}(t,L)=I(E_{z,i}\leq t)\int_0^L S_1(u)\int_0^u\frac{d\mathcal{M}_{z,i}(x)I(t-E_{z,i}\geq x)}{w_z(x,t)}du=\int_0^L\frac{A_z(u,L)}{w_z(u,t)}d\mathcal{M}_{z,i}(u)I(t-E_{z,i}\geq u),
$$

which is (20) of the main paper, where $A_z(u,L)=\int_u^L S_z(x)\,dx$, and the second equality follows by a change of variables and absorbing $I(E_{z,i}\leq t)$ into $I(t-E_{z,i}\geq u)$.

Variance and covariance of influence functions. From (20) of the main paper and using standard martingale results,

$$
\begin{aligned}
\text{var}\{IF_{R,z}(t_j,L_j)\}&=E\left\{\int_0^{L_j}\frac{A_z^2(u,L_j)}{w_z^2(u,t_j)}I(U_{z,i}\geq u,t_j-E_{z,i}\geq u)\lambda_z(u)du\right\}\\
&=\left\{\int_0^{L_j}\frac{A_z^2(u,L_j)}{w_z(u,t_j)}\lambda_z(u)du\right\},
\end{aligned}
$$

which is (22) of the main paper. Similarly, using standard counting process methods,

$$
\begin{aligned}
\text{cov}\{IF_{R,z,i}(t_j,L_j),IF_{R,z,i}(t_k,L_k)\}&=E\left\{\int_0^{L_j}\frac{A_z(u,L_j)A_z(u,L_k)}{w_z(u,t_j)w_z(u,t_k)}I(T_{z,i}\geq u,t_j-E_{1,i}\geq u)\lambda_z(u)\,du\right\}\\
&=\int_0^{L_j}\frac{A_z(u,L_j)A_z(u,L_k)}{w_z(u,t_k)}\lambda_z(u)\,du,
\end{aligned}
$$

which is (24) of the main paper.

E | IMPLEMENTATION USING THE LDBOUNDS PACKAGE

As noted in Section 5 of the main paper, we describe how we obtained the α-spending function and stopping boundaries in the simulations reported in Sections 4.2 and 4.3 and in the application to Study C9710 in Section 6 of the main paper using R and the `ldbounds` package, exemplifying how an analyst can carry out these tasks in practice. In both cases we took the overall significance level to be $\alpha = 0.05$.

In the simulations, where $K = 5$, with potential interim analysis times $t = (t_1, \ldots, t_5) = (1.5, 1.75, 2.0, 2.5, 3.0)$, we explicitly specified the α-spending function as $(\alpha_1, \ldots, \alpha_5) = (0.05, 0.1, 0.4, 0.7, 1) \times 0.05$, which we defined through the following.

```
> # alpha spending function
> alpha <- c(0.05,0.1,0.4,0.7,1)*0.05
> # alpha accumulated at each analysis
> diffalpha <- c(alpha[1],diff(alpha))
```

For use with the function `ldBounds()` in the `ldbounds` package to obtain stopping boundaries, at the jth interim analysis at time t_j, using $j = 3$ for illustration, we defined the following quantities and function and then called `ldBounds()` to obtain the stopping boundary ζ_3 to which $|\mathbb{T}_3|$ would be compared.

```
> # information at each time to time j
> info <- c(info1,info2,info3)
> j <- 3
> # alpha spending function for input to ldBounds, takes
> # j and info to be globally defined, as this function
> # can have only a single argument
> alphspend <- function(t){
     gg <- 0
     for (jj in 1:j){
     gg <- gg+diffalpha[jj]*(t>=info[jj])}
     return(gg)
 }
> # the stopping boundary for a two-sided test to which the
> # absolute value of the standardized test statistic is compared
> # is the element of bd$upper corresponding to the 3rd (jth) analysis
> bd <- ldBounds(t=info,iuse=5,asf=alphspend,alpha=0.05,sides=2)
> zeta.j <- bd$upper[j]
```

In the retrospective analysis of Study C9710, with $K = 16$ potential interim analysis times $t = (t_1, \ldots, t_{16}) = (560, 746, 933, 1120, 1310, 1490, 1680, 1870, 2050, 2240, 2430, 2610, 2800, 2990, 3170, 3360)$ days, for two-sided tests at each analysis time, we used the function `ldBounds()` in the `ldbounds` package to obtain the O'Brien Fleming α-spending function corresponding to the time since start of enrollment for each of the $K = 16$ planned analysis times as described in Section 6 as follows.

```
> t.interim <- c(560,746,933,1120,1310,1490,1680,1870,2050,2240,2430,2610,2800,
+ 2990,3170,3360)
> alpha.func <- ldBounds(t=t.interim/t.interim[16],iuse=1,alpha=0.05,sides=2)
> # alpha spending function
> alpha <- alpha.func$exit.pr
> round(alpha,8)
```

```
 [1] 0.00000008 0.00000393 0.00004209 0.00020701 0.00066221 0.00152609 0.00305065
 [8] 0.00532078 0.00822133 0.01209678 0.01679566 0.02197260 0.02815077 0.03499903
[15] 0.04204316 0.05000000
> # alpha accumulated at each analysis
> diffalpha <- alpha.func$diff.pr
```

For use with the function `ldBounds()` in the `ldbounds` package to obtain stopping boundaries, at the jth interim analysis at time t_j, we then proceeded identically as above, repeated here for definiteness using $j = 3$ for illustration, to obtain the stopping boundary ζ_3 to which $|\mathbb{T}_3|$ would be compared.

```
> # information at each time to time j
> info <- c(info1,info2,info3)
> j <- 3
> # alpha spending function for input to ldBounds, takes
> # j and info to be globally defined, as this function
> # can have only a single argument
> alphspend <- function(t){
     gg <- 0
     for (jj in 1:j){
     gg <- gg+diffalpha[jj]*(t>=info[jj])}
     return(gg)
 }
> # the stopping boundary for a two-sided test to which the
> # absolute value of the standardized test statistic is compared
> # is the element of bd$uppper corresponding to the 3rd (jth) analysis
> bd <- ldBounds(t=info,iuse=5,asf=alphspend,alpha=0.05,sides=2)
> zeta.j <- bd$upper[j]
```

Note that in the above, for two-sided tests, we obtain symmetric boundaries, and thus it suffices to use the values in `bd$upper` as ζ_j. See the documentation for the `ldbounds` package for more information.

REFERENCES

1. Tsiatis AA, Mehta C. On the inefficiency of the adaptive design for monitoring clinical trials. *Biometrika.* 2003;90:367–378.
